# Supplementary material for: Reversible interconversion between methanol-diamine and diamide for hydrogen storage based on manganese catalyzed (de)hydrogenation
Source: Nat Commun. 2020 Jan 30;11:591. doi: 10.1038/s41467-020-14380-3 (PMC6992753; doi:10.1038/s41467-020-14380-3)
Supplement: Supplementary file 1 — Supplementary Information [file 41467_2020_14380_MOESM1_ESM.pdf]

Supplementary Information

Supplementary figures

Supplementary Figure 1. Synthesis of 2-(diphenylphosphaneyl)ethan-1-amine

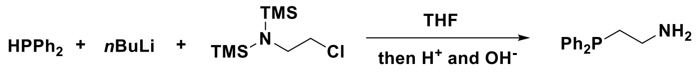

Supplementary Figure 2. Synthesis of **L-II**

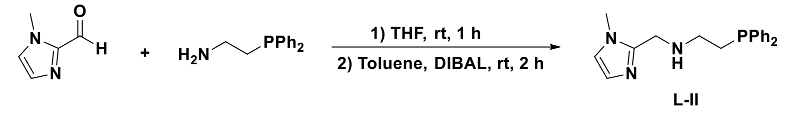

Supplementary Figure 3. Synthesis of **L-VI**

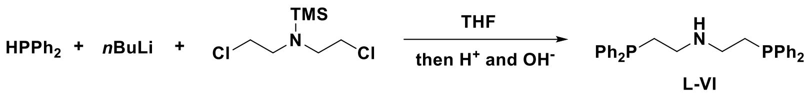

Supplementary Figure 4. Synthesis of **L-VII**

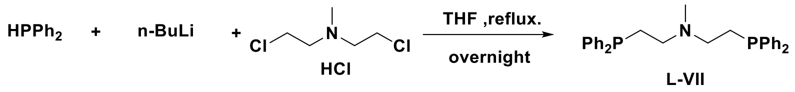

Supplementary Figure 5. Synthesis of **[Mn]-II**

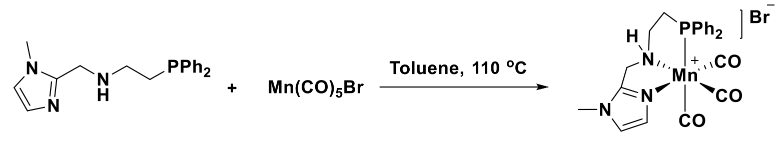

L-II **[Mn]-II**

Supplementary Figure 6. Synthesis of **[Mn]-VI**

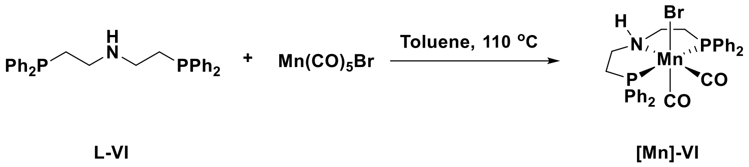

Supplementary Figure 7. Synthesis of **[Mn]-VII**

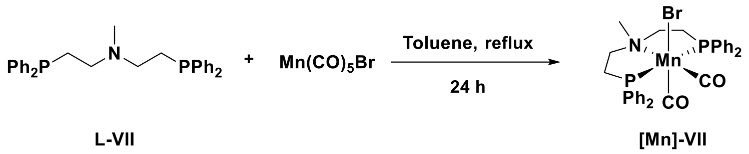

Supplementary Figure 8. Crystal structure of **[Mn]-VII**

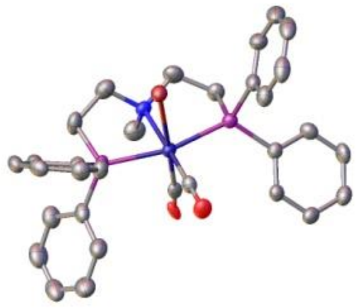

¹H NMR and GC analysis for the dehydrogenation coupling of *N,N'*-dimethylethylenediamine and methanol (Table 2, entry 11)

Supplementary Figure 9. ¹H NMR (400 MHz, $CDCl_3$) spectrum of (Table 2, entry 11)

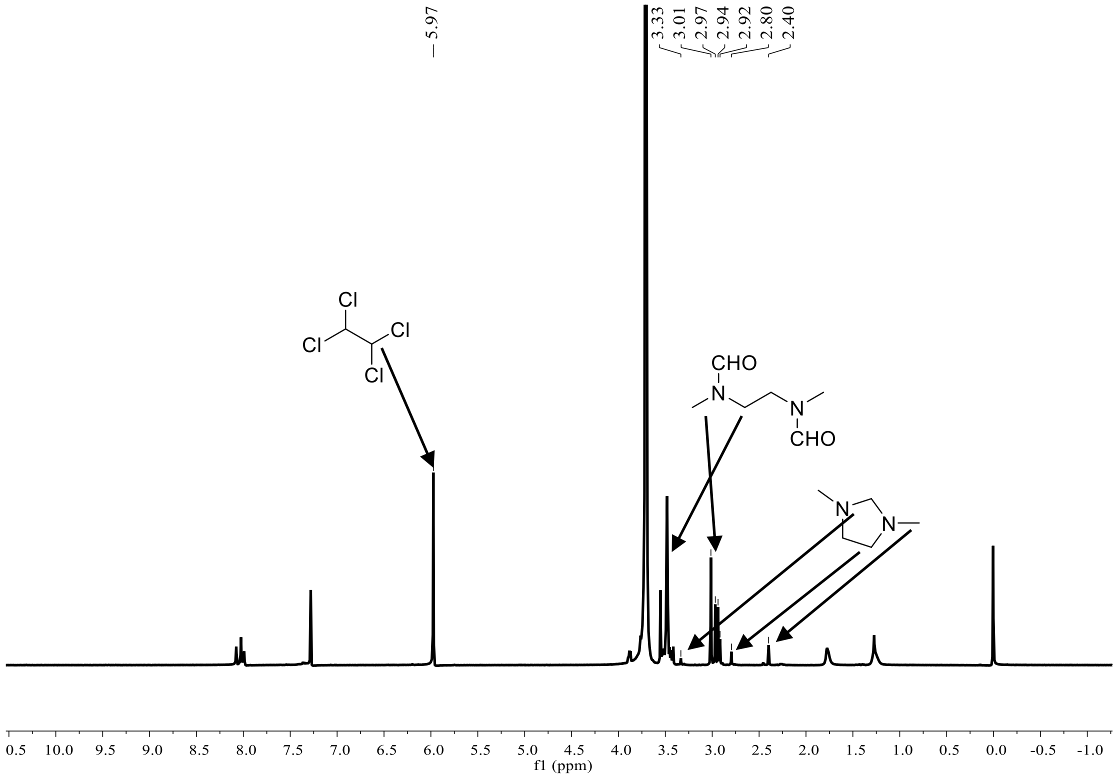

Supplementary Figure 10.GC analysis of (Table 2, entry 11)

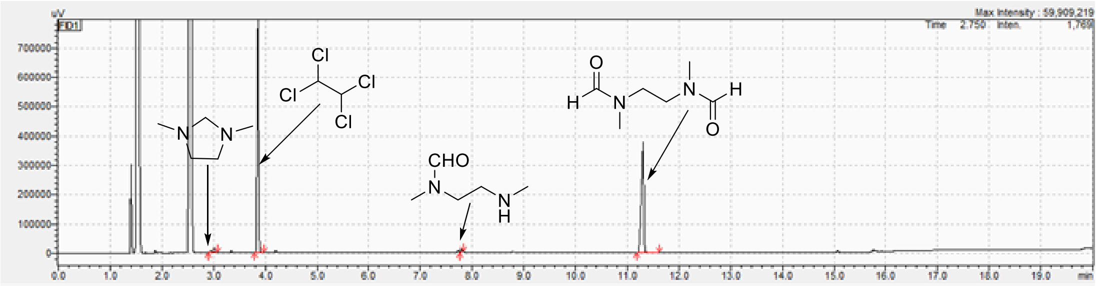

结果视图 - 峰表

峰表 | 化合物 | 组 | 校准曲线

峰号	保留时间	面积	高度	标记	浓度	浓度单位	化合物ID号	化合物名	面积%
1	2.935	31939	6975	M	1.071				1.071
2	3.858	1687826	871723	V	56.572				56.572
3	7.792	20866	13422	M	0.699				0.699
4	11.309	1242863	373862		41.658				41.658
总计		2983494	1265982		100.000				100.000

^{1}H NMR analysis for the hydrogenation of *N*,*N*'-(Ethane-1,2-diyl)bis(*N*-methylformamide) (Table 3 entry 4)

Supplementary Figure 11.^{1}H NMR (400 MHz, $CDCl_3$) spectrum of (Table 3, entry 4)

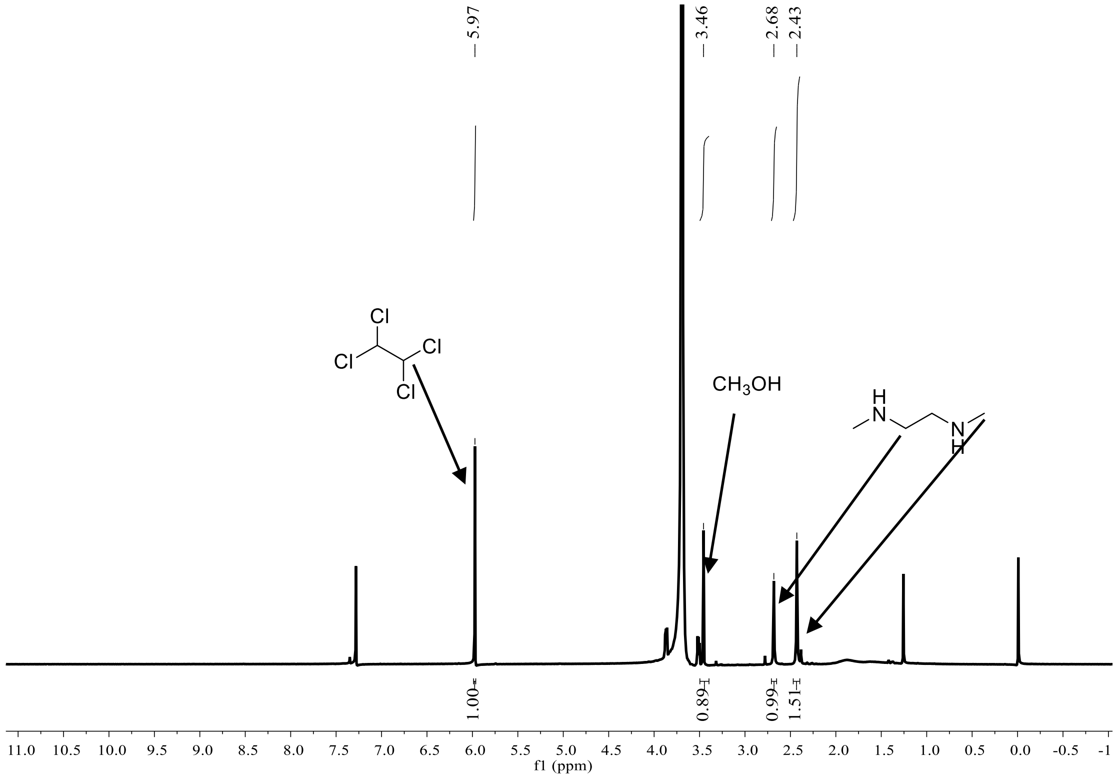

Supplementary Figure 12. Gas collector

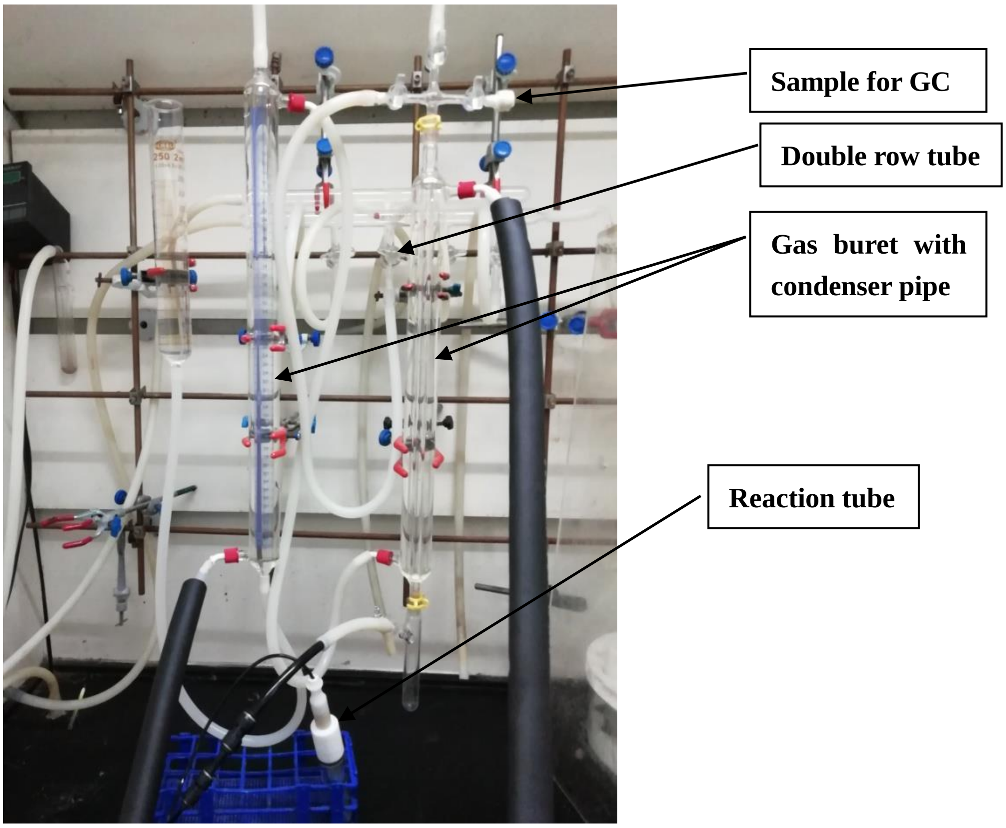

Supplementary Figure 13. GC analysis of the gas phase (the first 2 h for table 2, entry 11)

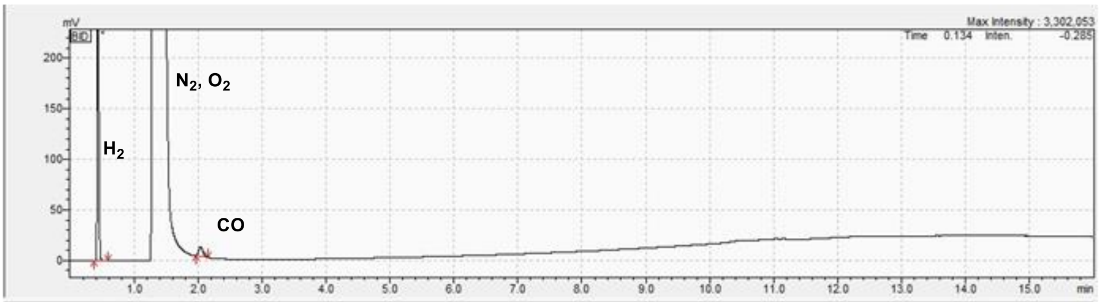

结果视图 - 峰表

峰表 | 化合物 | 组 | 校准曲线

峰号	保留时间	面积	高度	标记	浓度	浓度单位	化合物ID号	化合物名	面积%	S/N
1	0.427	550479	343130		14932.155	ppm	1	H2	92.478	2.84
2	2.032	44778	9477	M	281.847	ppm	2	CO	7.522	0.08
总计		595257	352608		15214.001				100.000	

Purity of H_2 = 14932.155 / (14932.155 + 281.847) = 98.1%

Purity of CO = 1 – H_2 % = 1.9%

Supplementary Figure 14. GC analysis of the gas phase (the next 6 h for table 2, entry 11)

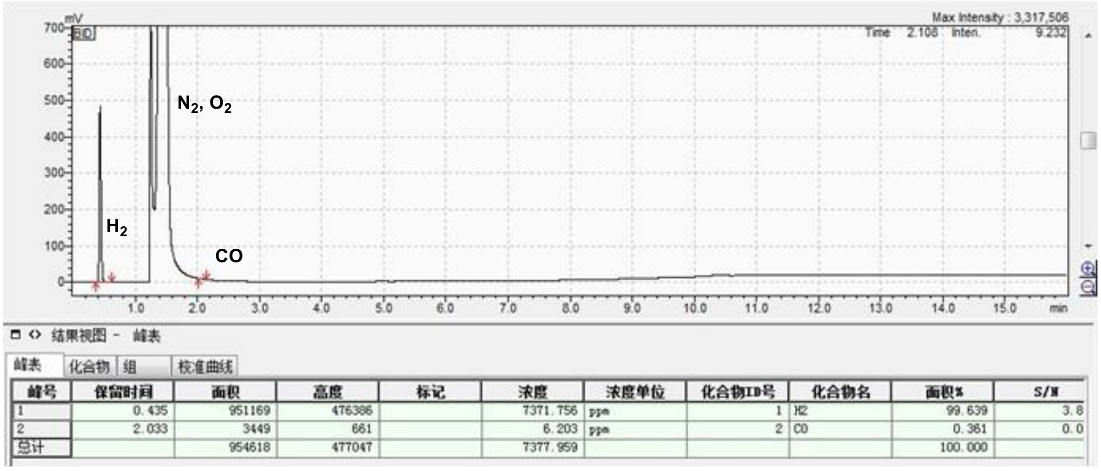

峰号	保留时间	面积	高度	标记	浓度	浓度单位	化合物ID号	化合物名	面积%	S/N
1	0.435	951169	476386		7371.756	ppm	1	H2	99.639	3.8
2	2.033	3449	661		6.203	ppm	2	CO	0.361	0.0
总计		954618	477047		7377.959				100.000	

Purity of H_2 = 7371.756 / (7371.756 + 6.203) = 99.9%

Purity of CO = 1 – H_2 % = 0.1%

Purity of H_2 for the whole reaction = [(16.4 * 0.981) + (7.6 * 0.999)] / (16.4 + 7.6) = 98.7%

Purity of CO for the whole reaction = 1 – H_2 % = 1.3%

Yield of H_2 for the whole reaction = [(16.4 + 7.6) *0.987] / 24.1 = 98.3%

Supplementary Figure 15. GC analysis of the gas phase (the first 2 h for table 2, entry 12)

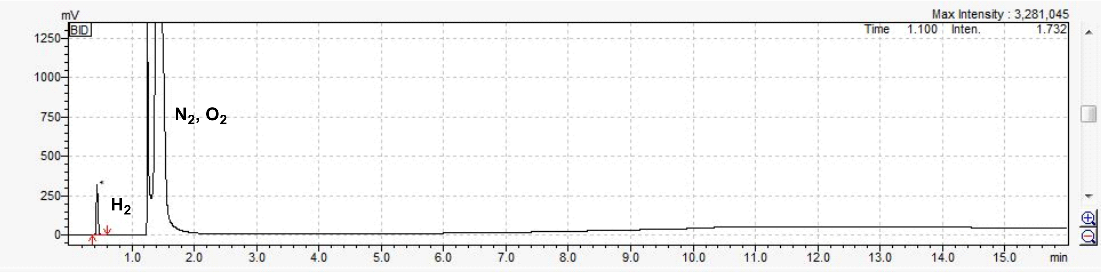

结果视图 - 峰表

峰表 | 化合物 | 组 | 校准曲线

峰号	保留时间	面积	高度	标记	浓度	浓度单位	化合物ID号	化合物名	面积%	S/N
1	0.442	607966	316455		4590.823	ppm	1	H2	100.000	2.59
总计		607966	316455		4590.823				100.000	

Purity of H_2 > 99.9%

Purity of CO (not detected)

Supplementary Figure 16. GC analysis of gas the phase (The next 6 h for table 2, entry 12)

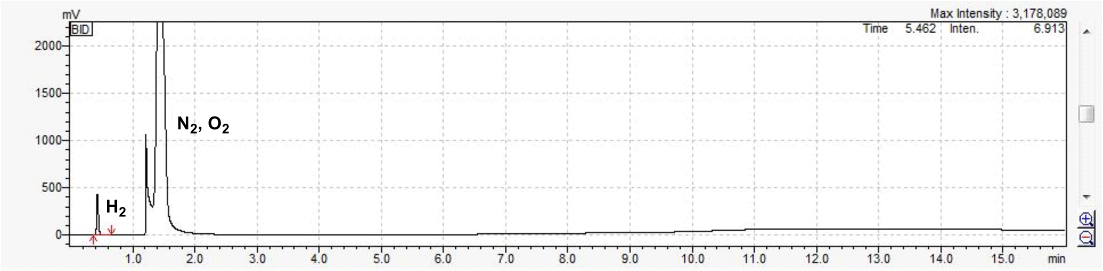

结果视图 - 峰表

峰表 | 化合物 | 组 | 校准曲线

峰号	保留时间	面积	高度	标记	浓度	浓度单位	化合物ID号	化合物名	面积%	S/N
1	0.433	827571	429286		6227.652	ppm	1	H2	100.000	3.76
总计		827571	429286		6227.652				100.000	

Purity of H_2 > 99.9%

Purity of CO (not detected)

Purity of H_2 for the whole reaction > 99.9%

Purity of CO for the whole reaction (not detected)

Yield of H_2 for the whole reaction = (10.6 + 11.0)/ 24.1 = 89.6%

Supplementary Figure 17. GC analysis of the gas phase (the first 4 h for fig 2)

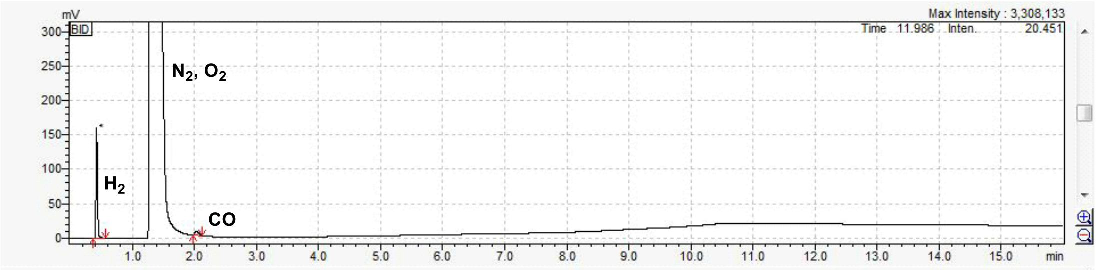

结果视图 - 峰表

峰表 | 化合物 | 组 | 校准曲线

峰号	保留时间	面积	高度	标记	浓度	浓度单位	化合物ID号	化合物名	面积%	S/N
1	0.426	254947	157102		6915.612	ppm	1	H2	91.473	1.2
2	2.037	23767	5300	M	149.597	ppm	2	CO	8.527	0.0
总计		278713	162401		7065.209				100.000	

Purity of H_2 = 6915.612 / (6915.612 + 149.597) = 97.9%

Purity of CO = 1 – H_2 % = 2.1%

Supplementary Figure 18. GC analysis of the gas phase (the next 6 h for fig 2)

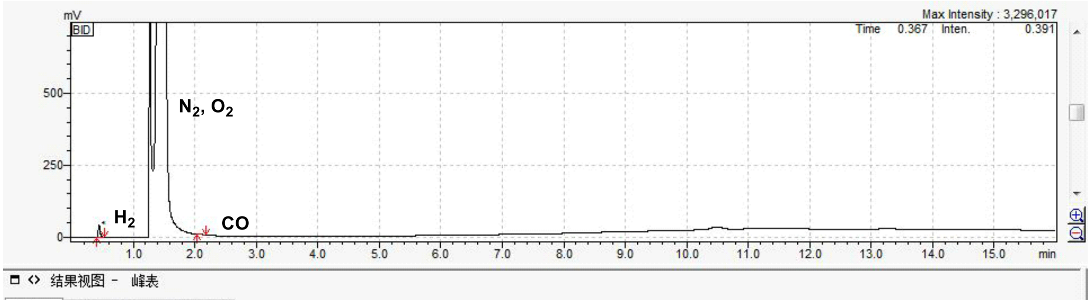

峰号	保留时间	面积	高度	标记	浓度	浓度单位	化合物ID号	化合物名	面积%	S/N
1	0.443	83065	41693		643.772	ppm	1	H2	94.108	0.3
2	2.060	5200	1175		9.352	ppm	2	CO	5.892	0.0
总计		88266	42868		653.125				100.000	

Purity of H_2 = 643.772 / (643.772 + 9.352) = 98.6%

Purity of CO = 1 – H_2 % = 1.4%

Purity of H_2 for the whole reaction = [(30.8 * 0.979) + (14.4 * 0.986)] / (30.8 + 14.4) = 98.1%

Purity of CO for the whole reaction = 1 – H_2 % = 1.9%

Yield of H_2 for the whole reaction = [(30.8 + 14.4) * 0.981]/ (24.1 * 2) = 92.0%

Supplementary Figure 19. Reversible interconversion between **1**/methanol and **2a** catalyzed by **Mn-IV** and **Mn-VI**

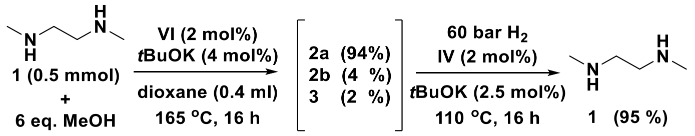

Supplementary Figure 20. Reversible interconversion between **1**/methanol and **2a** catalyzed by **Mn-VI**

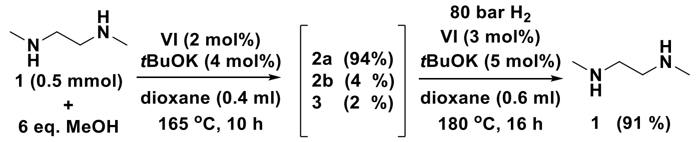

Supplementary Figure 21. Mn-catalyzed dehydrogenation of MeOH (Fig 4a)

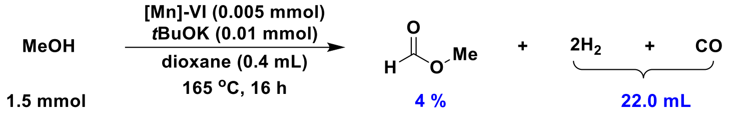

Supplementary Figure 22.GC analysis of the gas phase (Fig 4a)

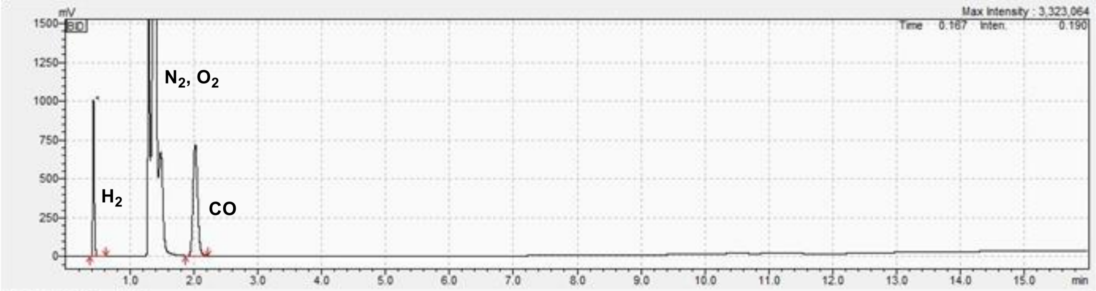

结果视图 - 峰表

峰表 | 化合物 | 组 | 校准曲线

峰号	保留时间	面积	高度	标记	浓度	浓度单位	化合物ID号	化合物名	面积%	S/N
1	0.424	1641523	994895		44527.511	ppm	1	H2	31.572	8.56
2	2.018	3557843	717481	M	22394.367	ppm	2	CO	68.428	6.17
总计		5199367	1712376		66921.877				100.000	

Purity of H_2 = 44527.511 / (44527.511 + 22394.367) = 66.5%

Purity of CO = 1 – H_2 % = 33.5%

$V(H_2)$ = 22.0 * 0.665 = 14.6 mL

V(CO) = 22.0 - $V(H_2)$ = 7.4 mL

Supplementary Figure 23.¹H NMR (400 MHz, CDCl₃) spectrum of Fig 4a

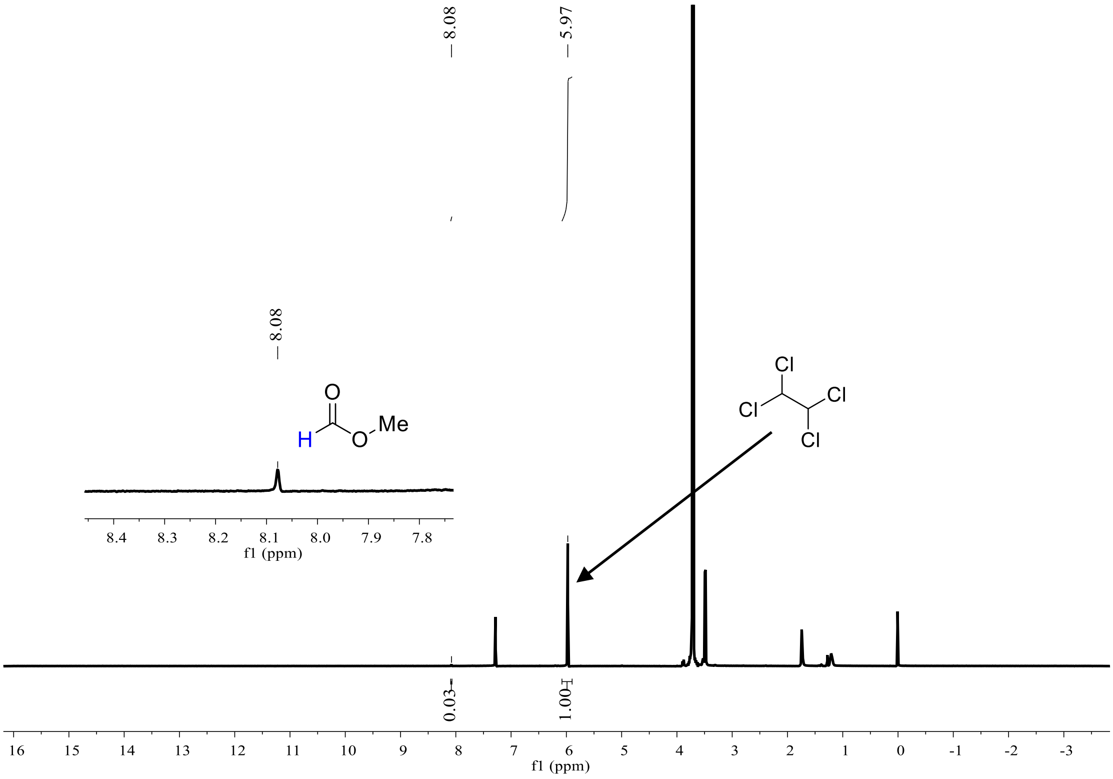

Supplementary Figure 24.^{1}H NMR (400 MHz, $CDCl_3$) spectrum of L-II

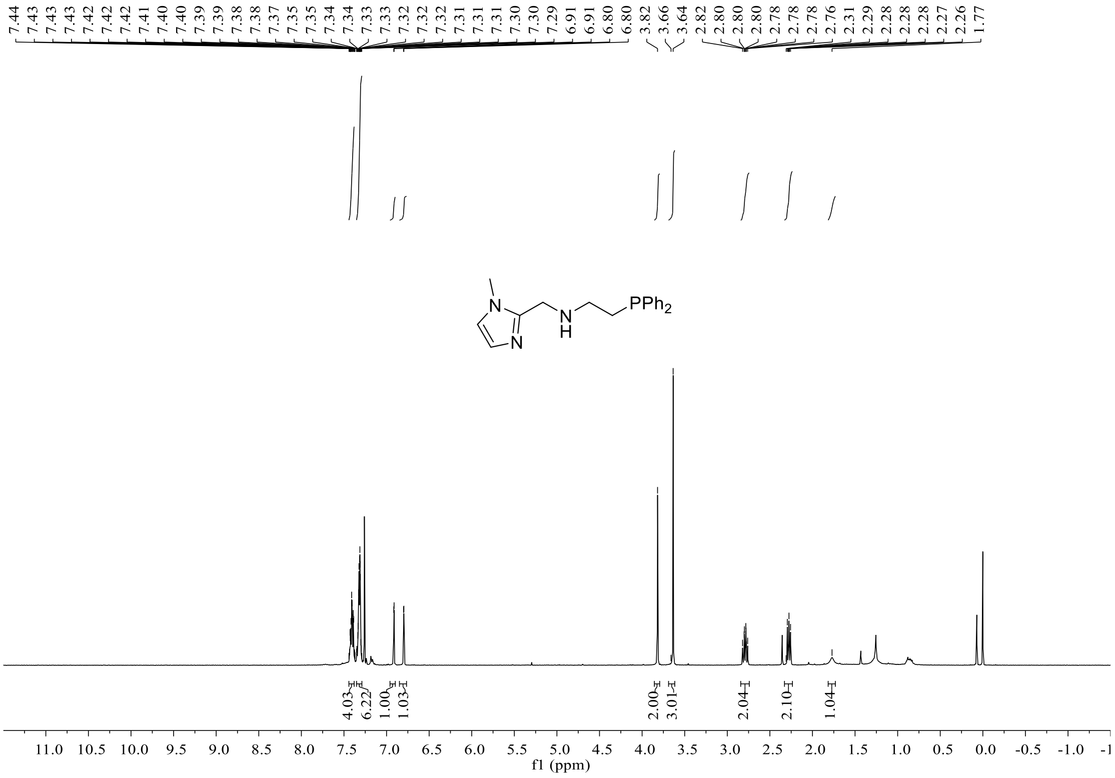

Supplementary Figure 25.^{13}**C NMR (100 MHz, CDCl$_3$) spectrum of L-II**

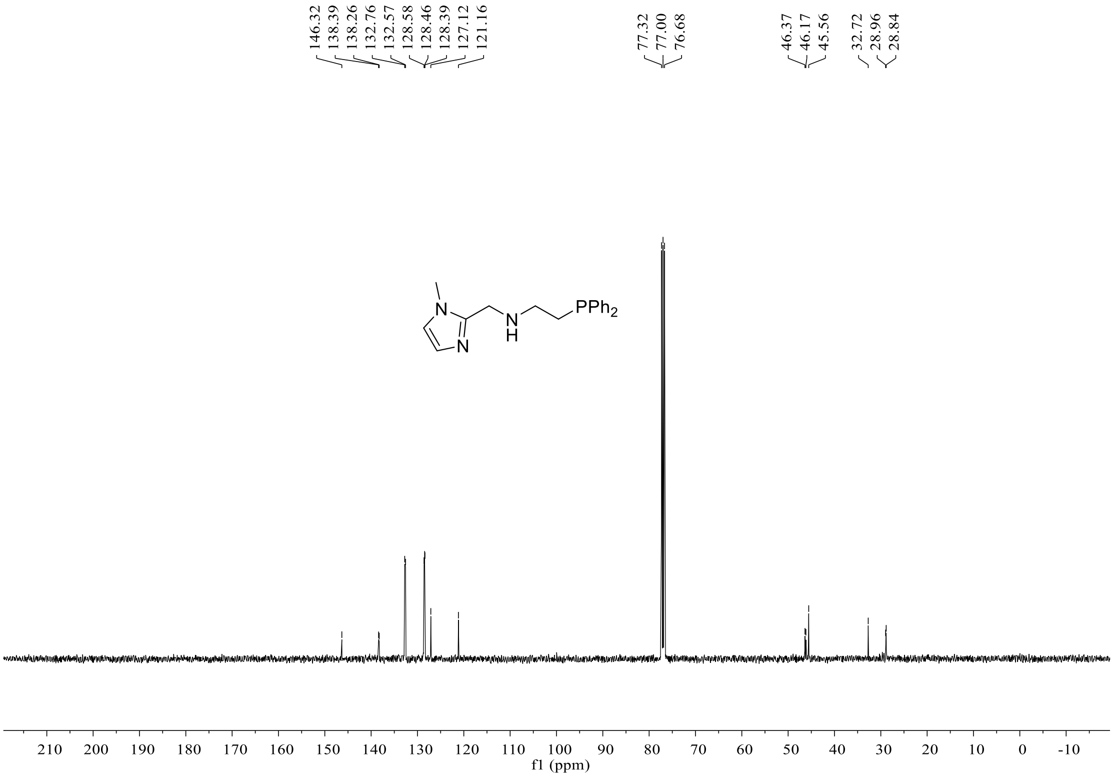

Supplementary Figure 26. ^{31}P **NMR (162 MHz,** $CDCl_3$**) spectrum of L-II**

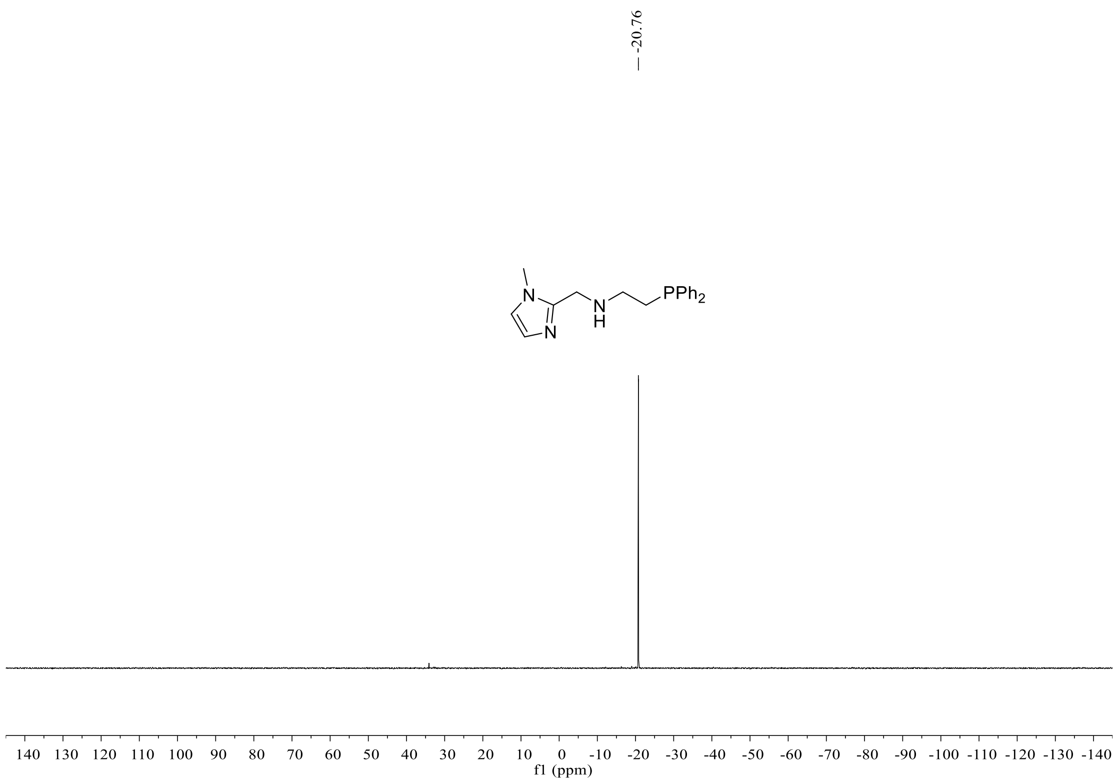

Supplementary Figure 27.^{1}H NMR (400 MHz, $CDCl_3$) spectrum of L-VI

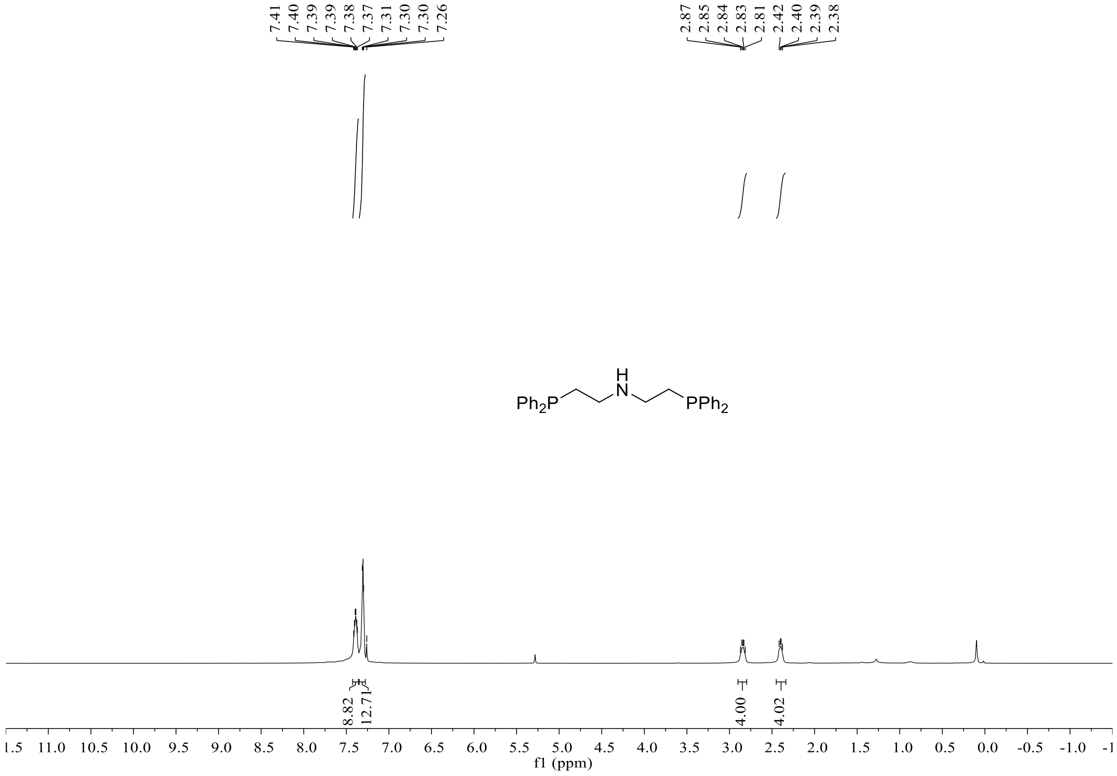

Supplementary Figure 28. ^{13}C NMR (100 MHz, CDCl$_3$) spectrum of L-VI

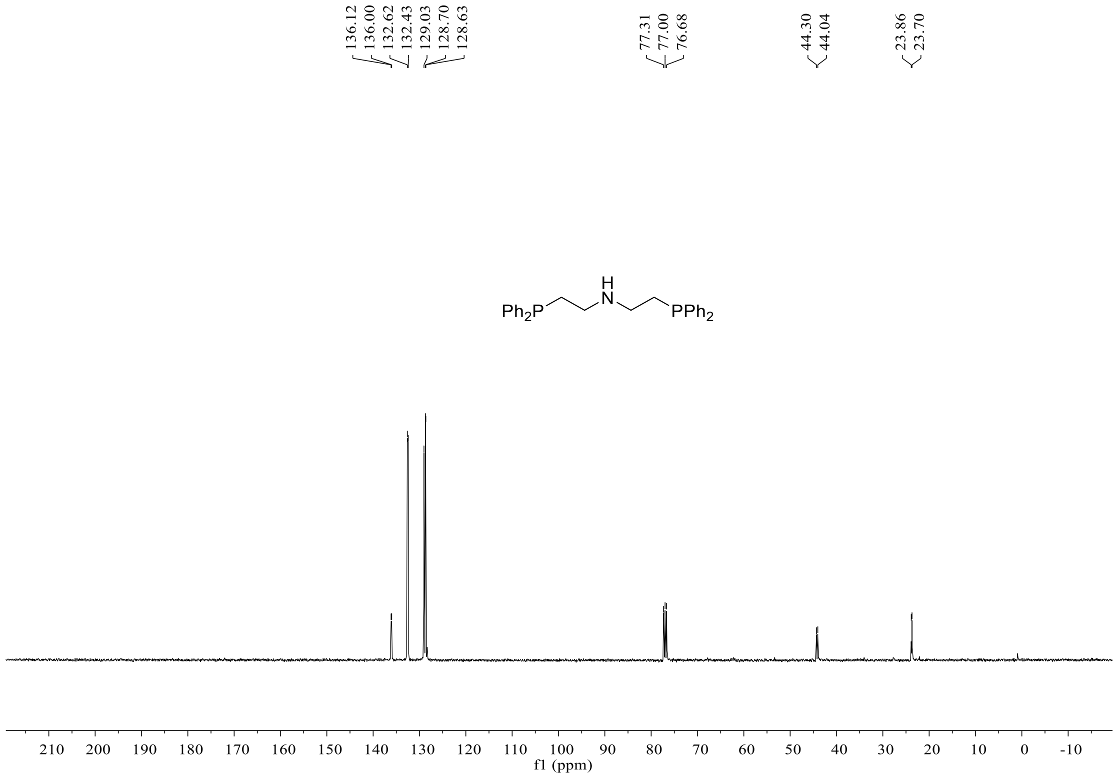

Supplementary Figure 29.^{31}P NMR (162 MHz, $CDCl_3$) spectrum of L-VI

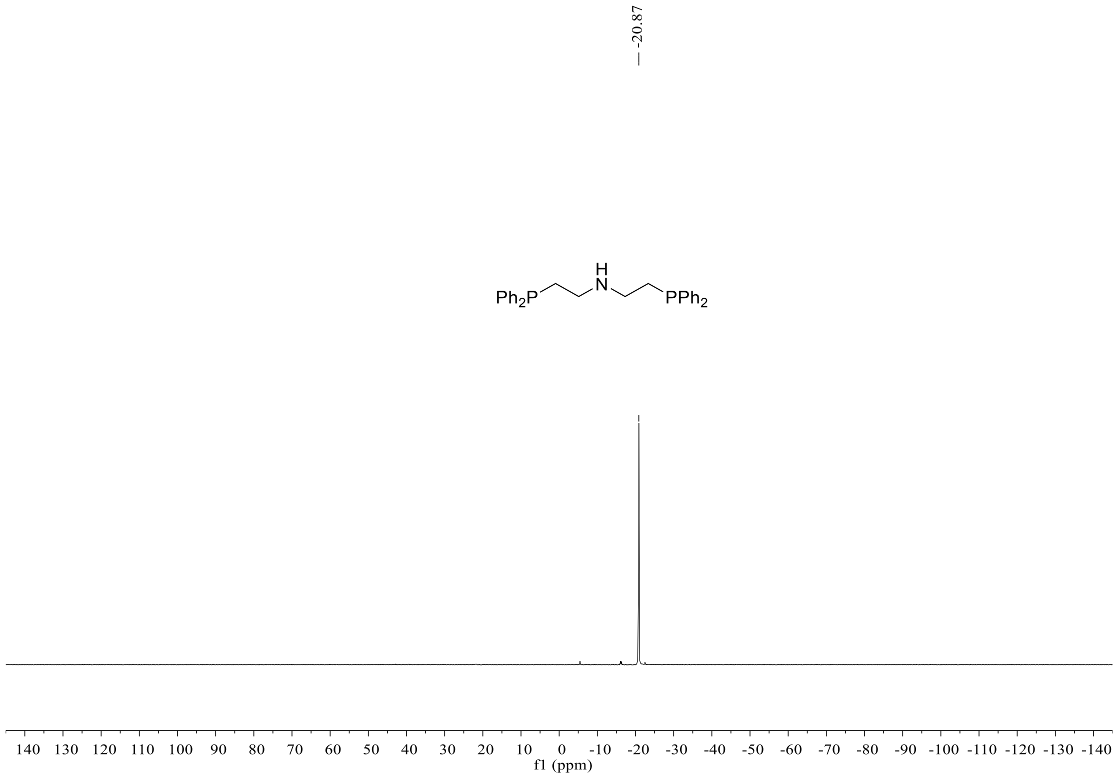

Supplementary Figure 30.^{1}H NMR (400 MHz, $CDCl_3$) spectrum of L-VII

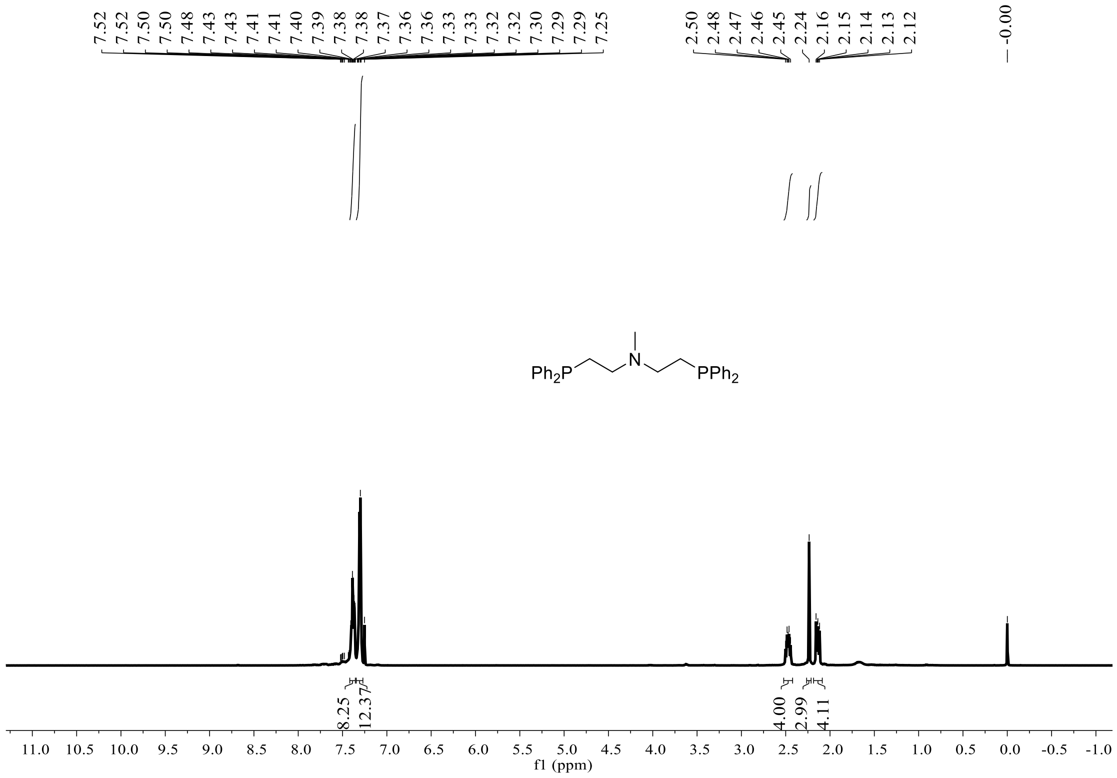

Supplementary Figure 31. ^{13}C **NMR (100 MHz,** $CDCl_3$**) spectrum of L-VII**

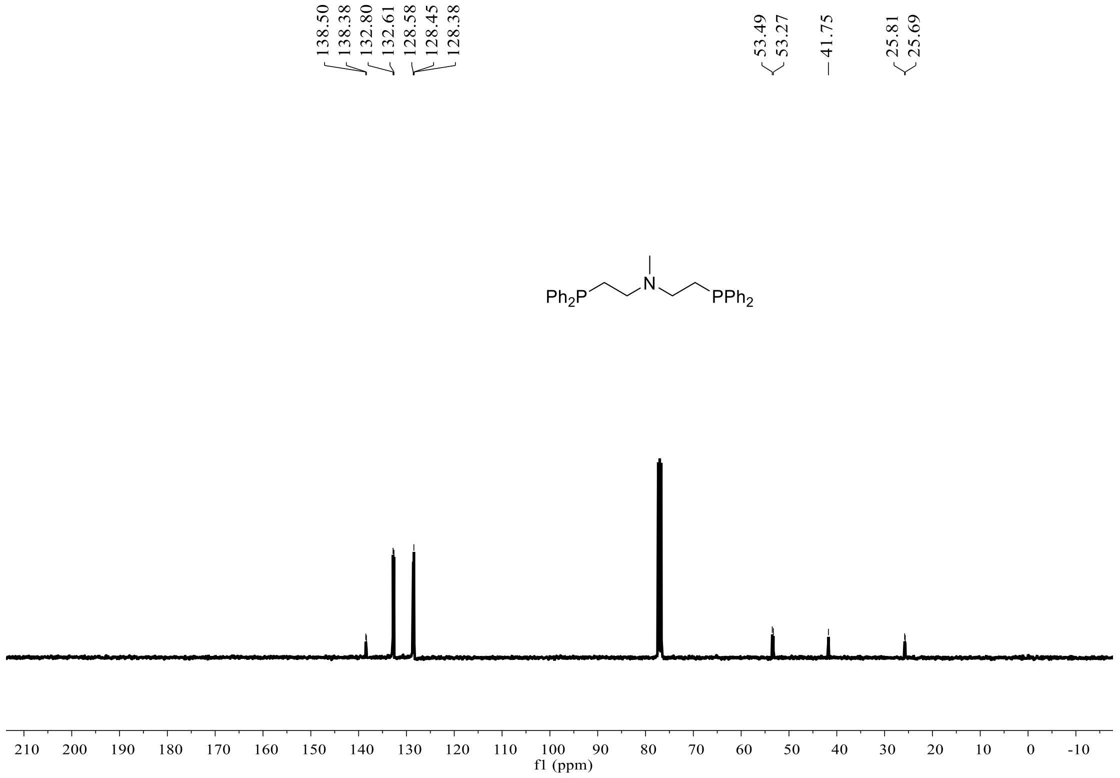

Supplementary Figure 32. ^{31}P **NMR (162 MHz,** $CDCl_3$**) spectrum of L-VII**

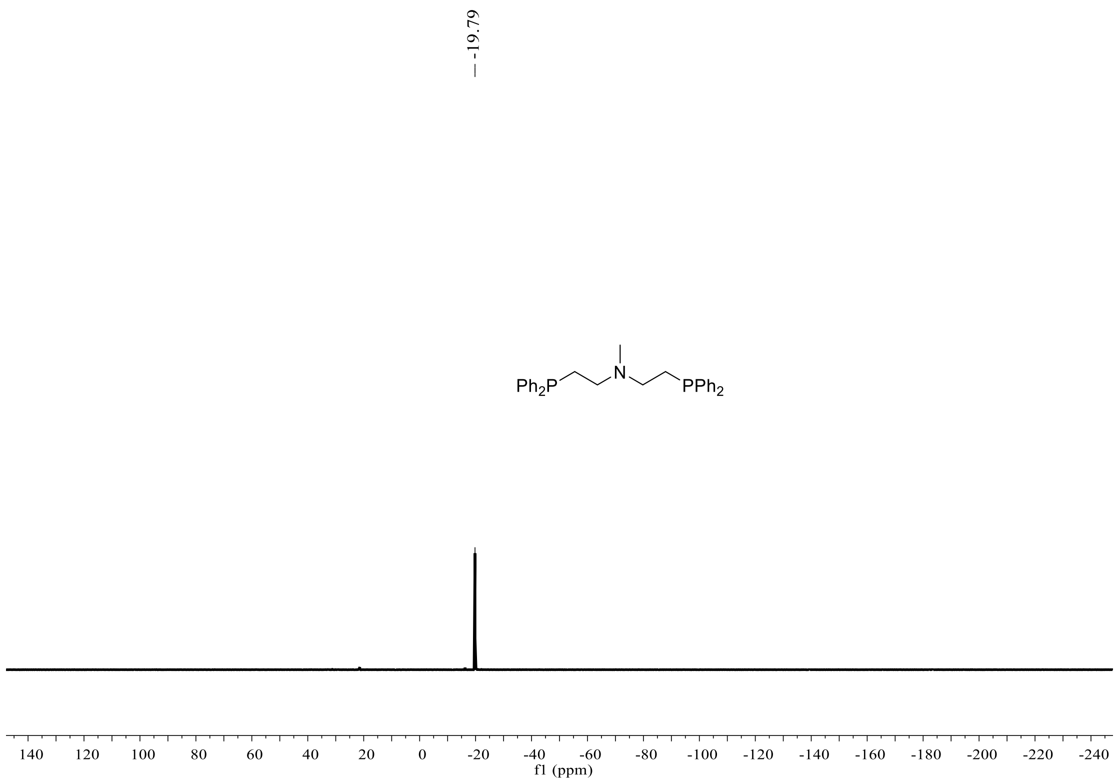

Supplementary Figure 33.^{1}H NMR (400 MHz, DMSO-d_6) spectrum of Mn-II

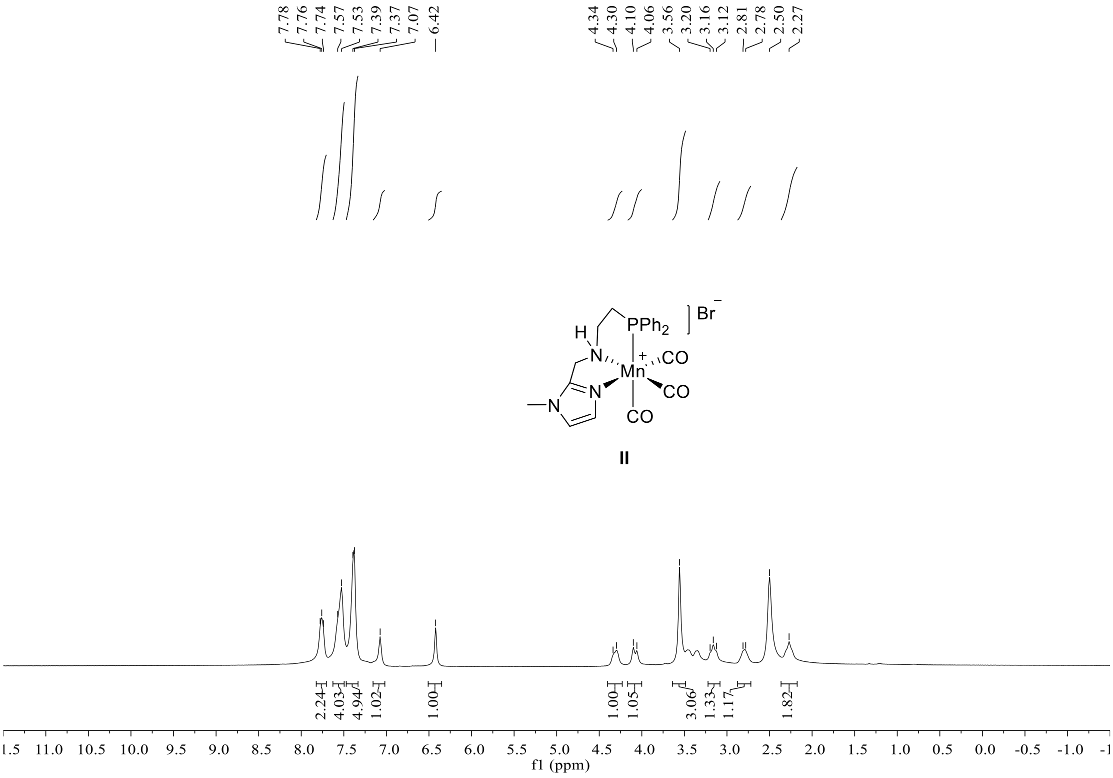

Supplementary Figure 34. ^{13}C NMR (100 MHz, DMSO-d_6) spectrum of Mn-II

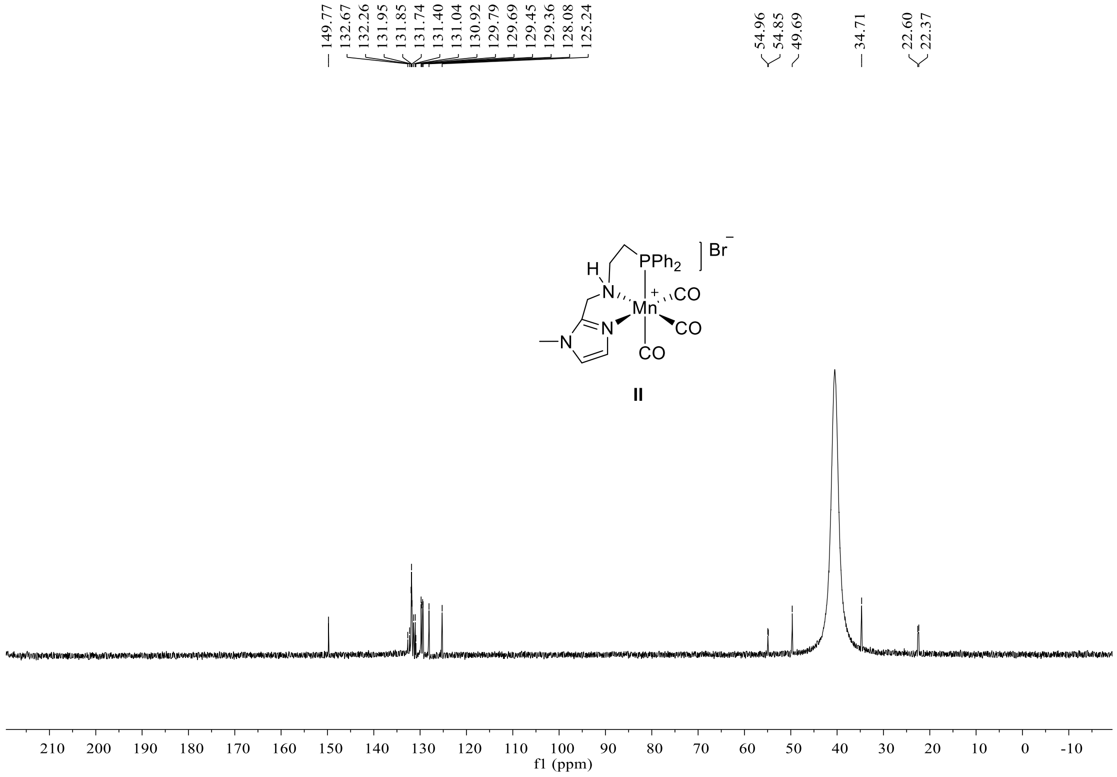

Supplementary Figure 35.^{31}P NMR (162 MHz, DMSO-d_6) spectrum of Mn-II

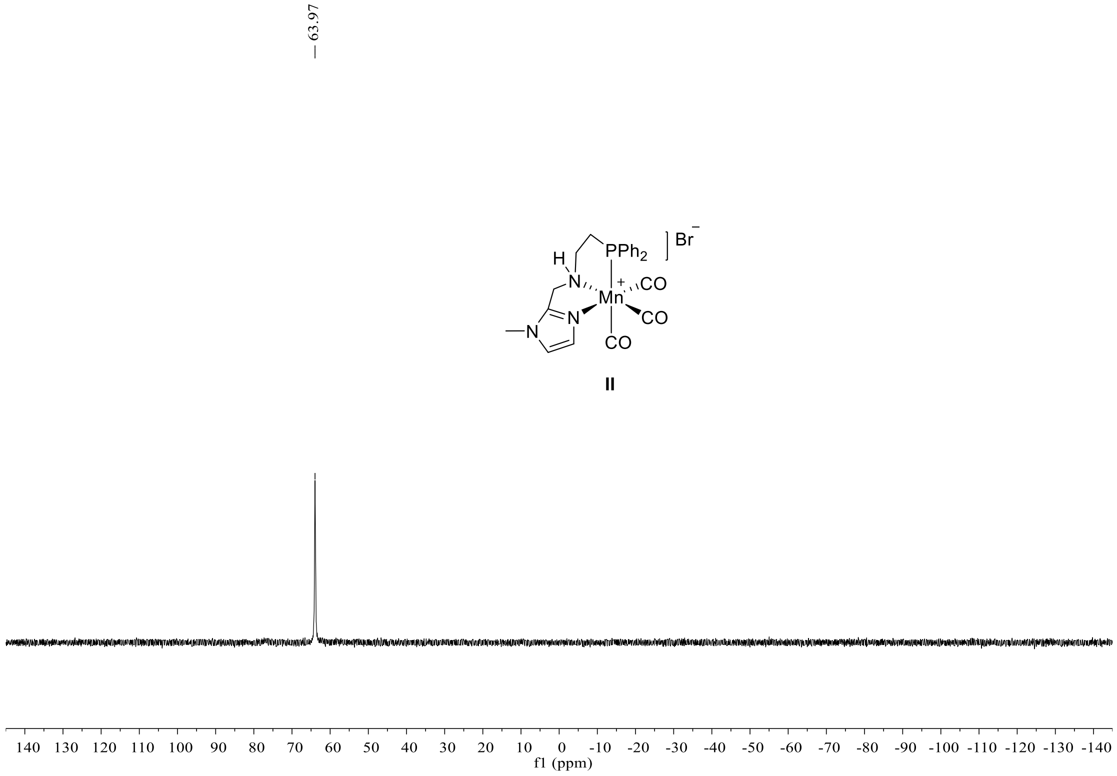

Supplementary Figure 36.[1]**H NMR (400 MHz, DCM-*d*$_2$) spectrum of Mn-VI**

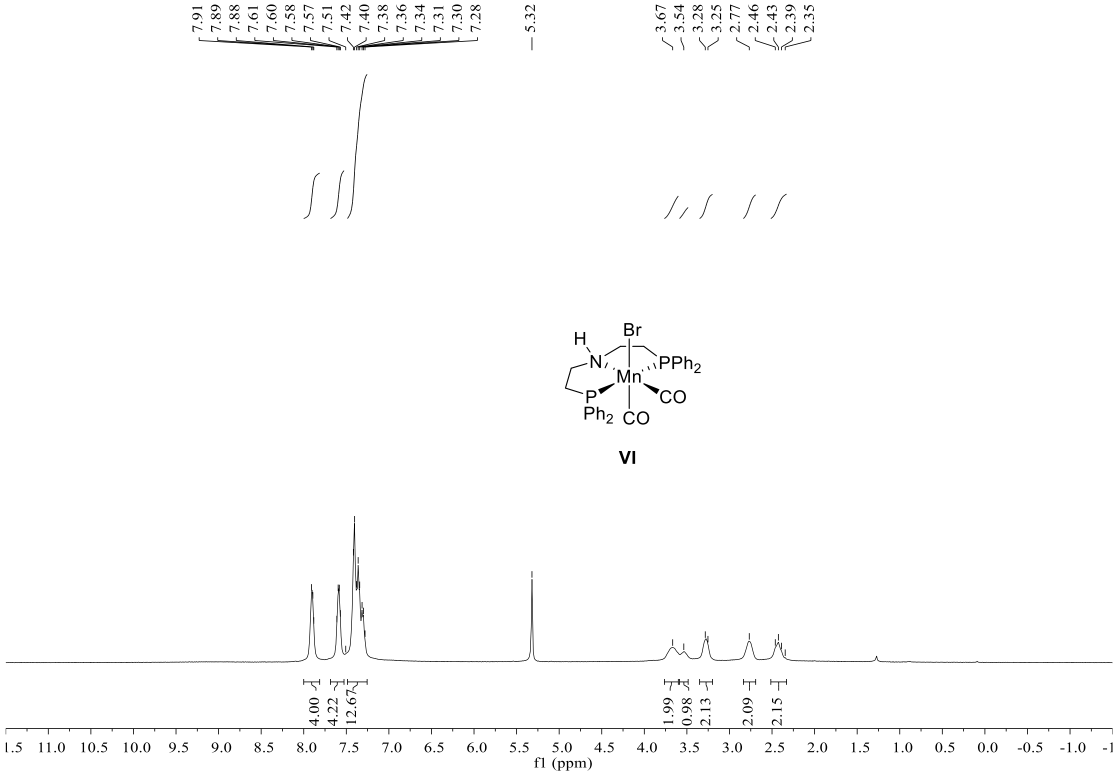

Supplementary Figure 37. ^{13}C **NMR (100 MHz, DCM-d_2) spectrum of Mn-VI**

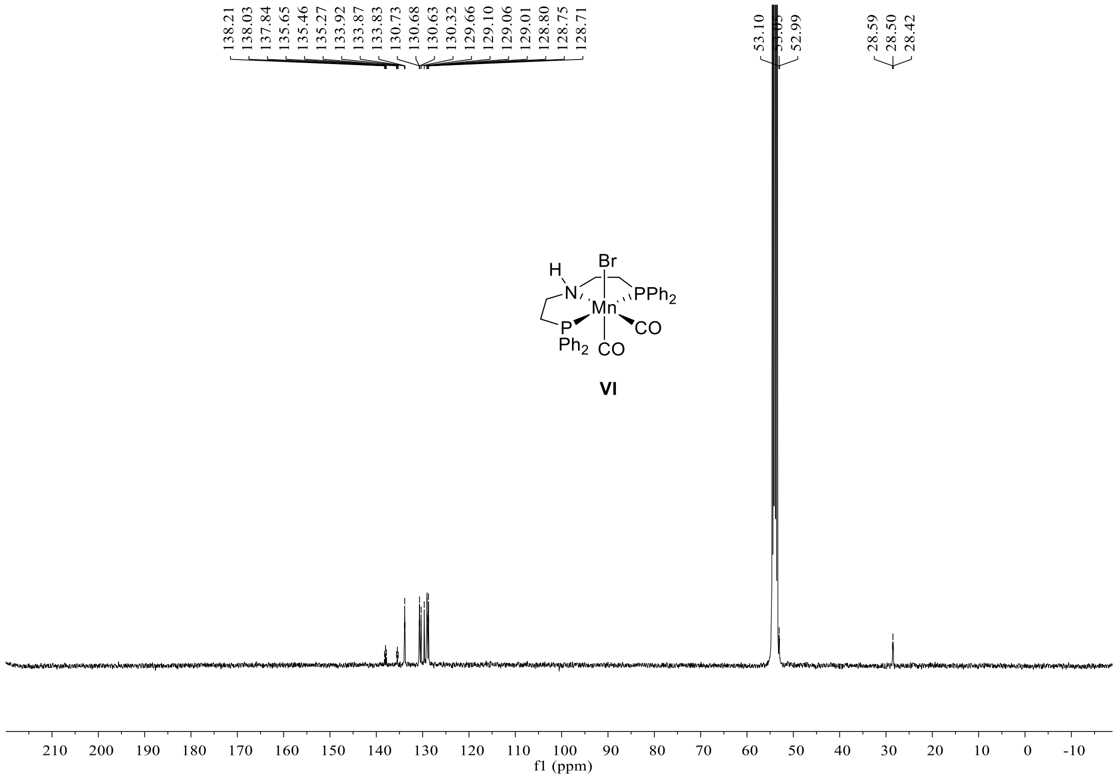

Supplementary Figure 38. ^{31}P NMR (162 MHz, DCM-d_2) spectrum of Mn-VI

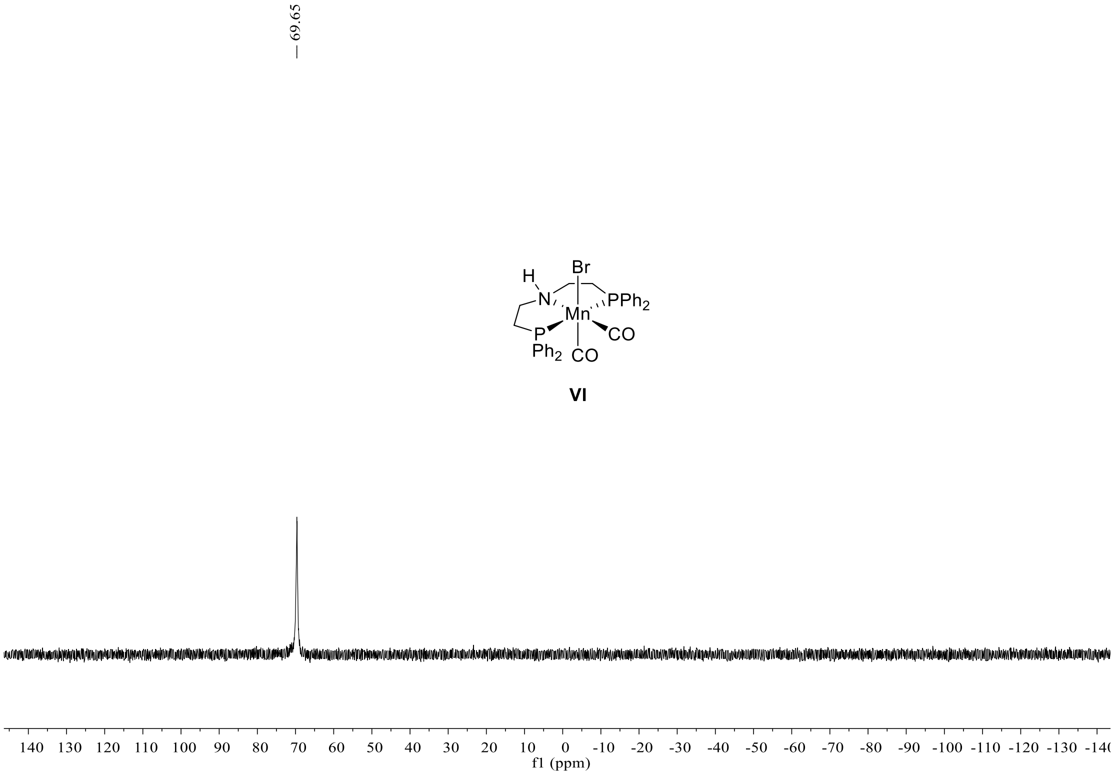

Supplementary Figure 39.^{1}H NMR (400 MHz, DCM-d_2) spectrum of Mn-VII

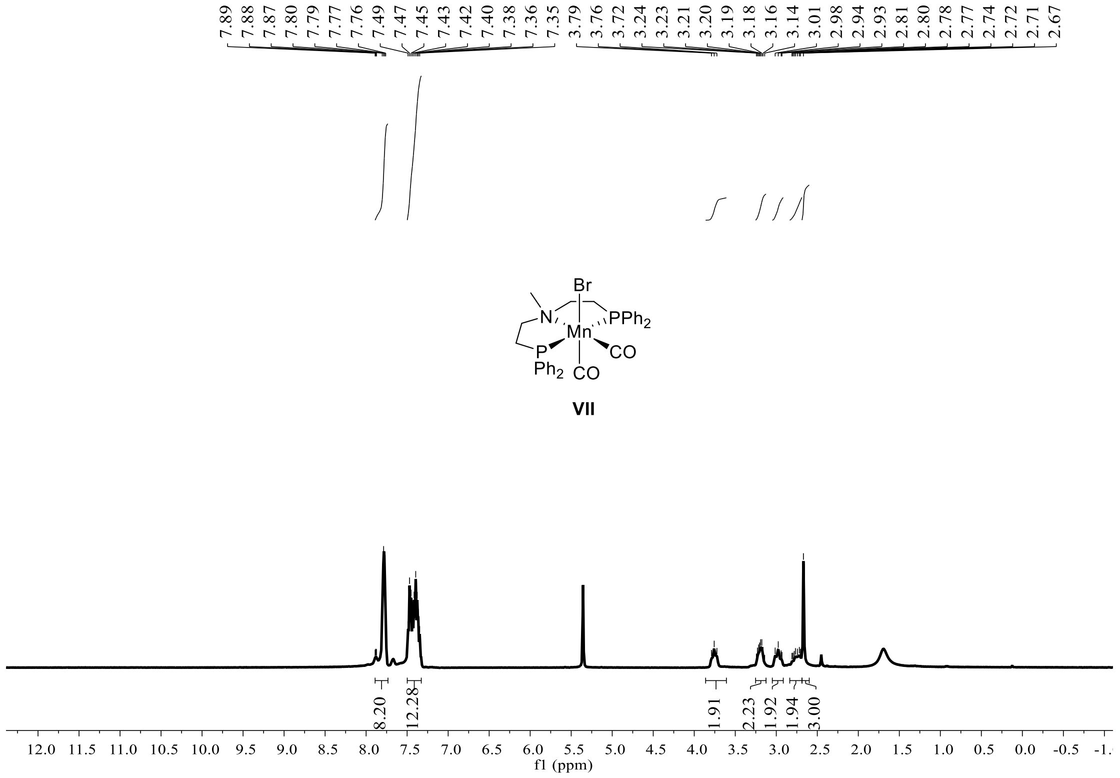

Supplementary Figure 40.^{13}C NMR (100 MHz, DCM-d_2) spectrum of Mn-VII

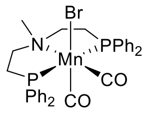

VII

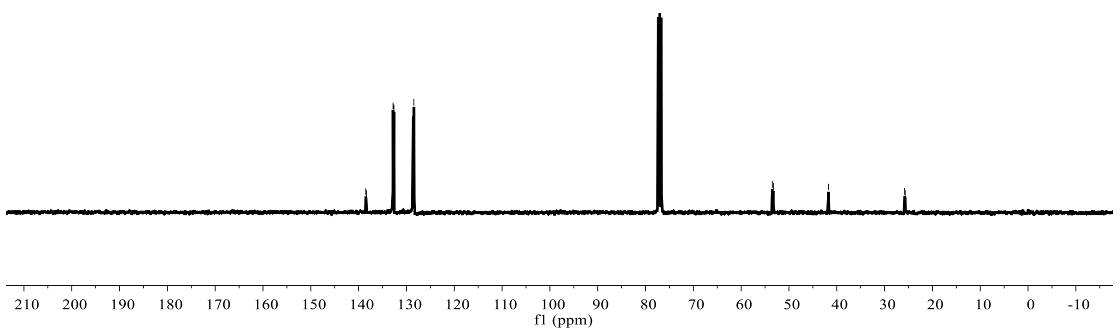

Supplementary Figure 41. ^{31}**P NMR (162 MHz, DCM-*d*$_2$) spectrum of Mn-VII**

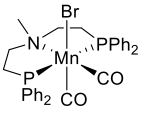

VII

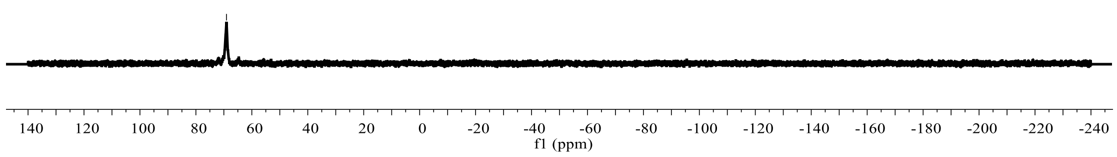

Supplementary Figure 42.^{1}H NMR (400 MHz, $CDCl_3$) spectrum of 2a

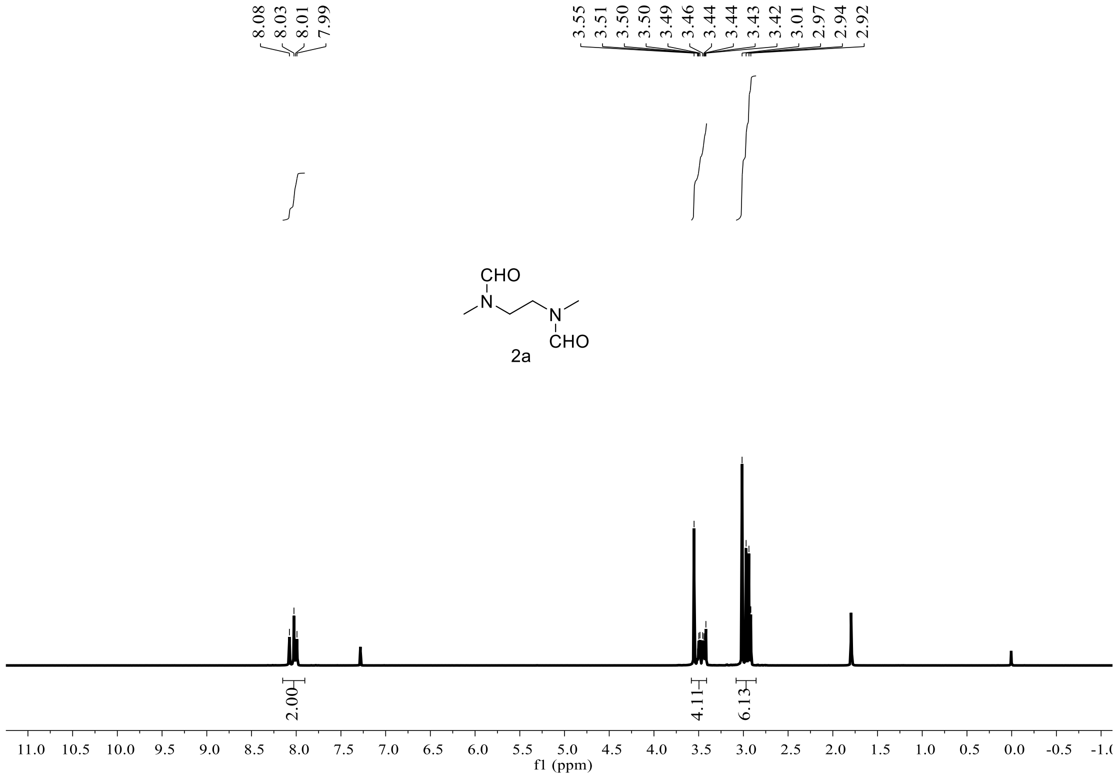

Supplementary Figure 43.^{13}C NMR (100 MHz, $CDCl_3$) spectrum of 2a

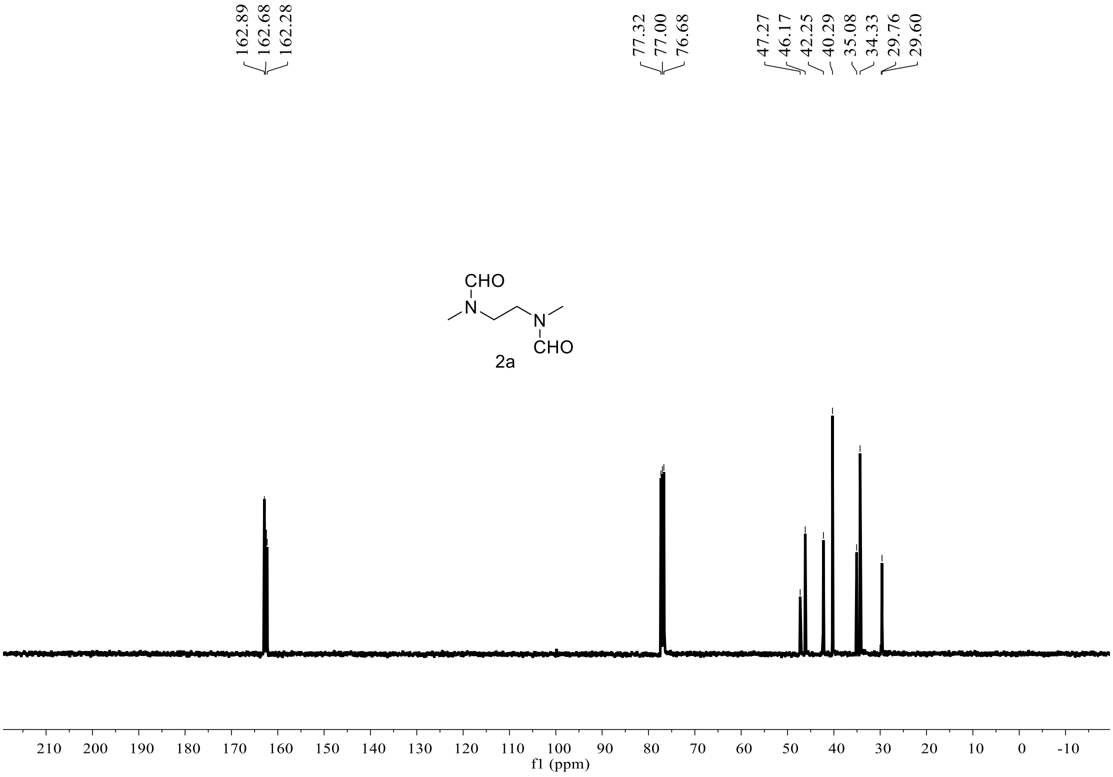

Supplementary Figure 44.^{1}H NMR (400 MHz, $CDCl_3$) spectrum of 2b

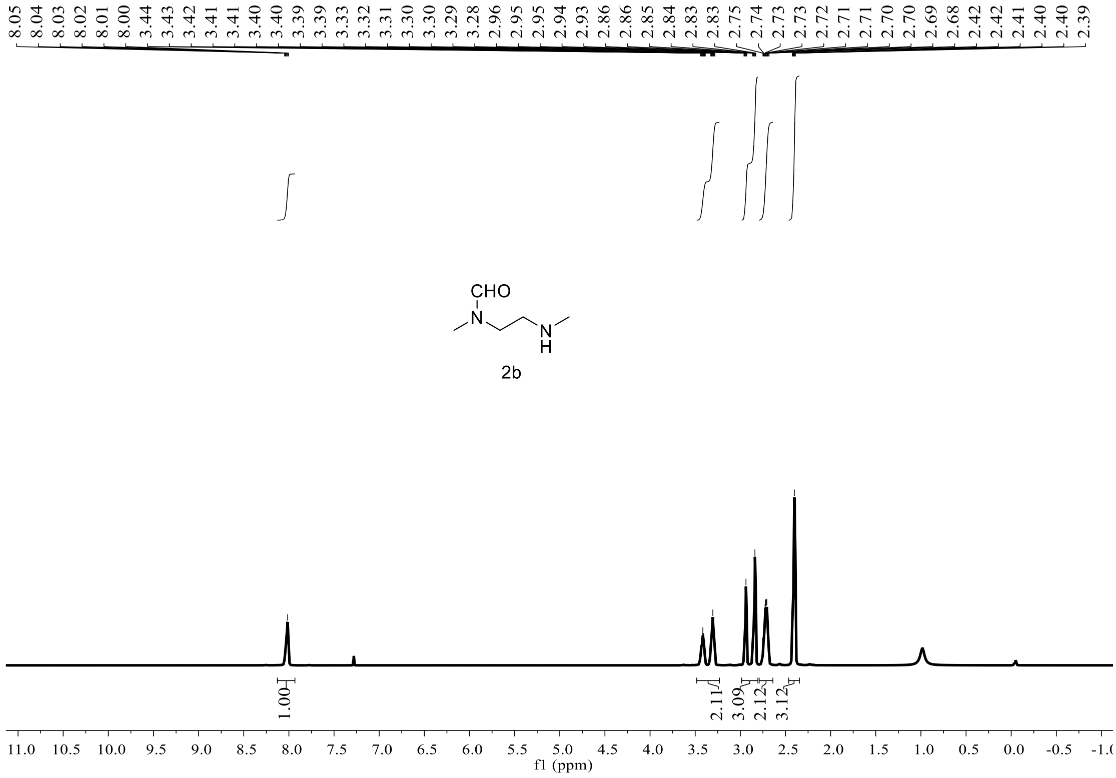

Supplementary Figure 45.^{13}C NMR (100 MHz, $CDCl_3$) spectrum of 2b

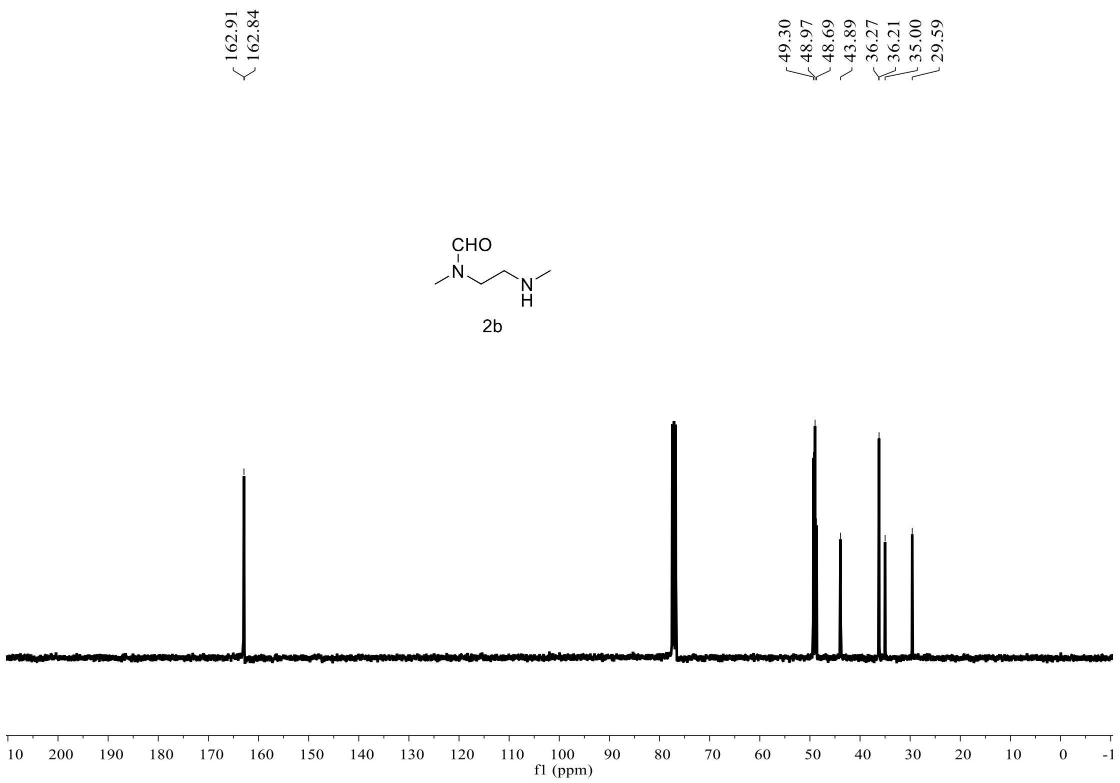

Supplementary Figure 46.^{1}H NMR (400 MHz, $CDCl_3$) spectrum of 3

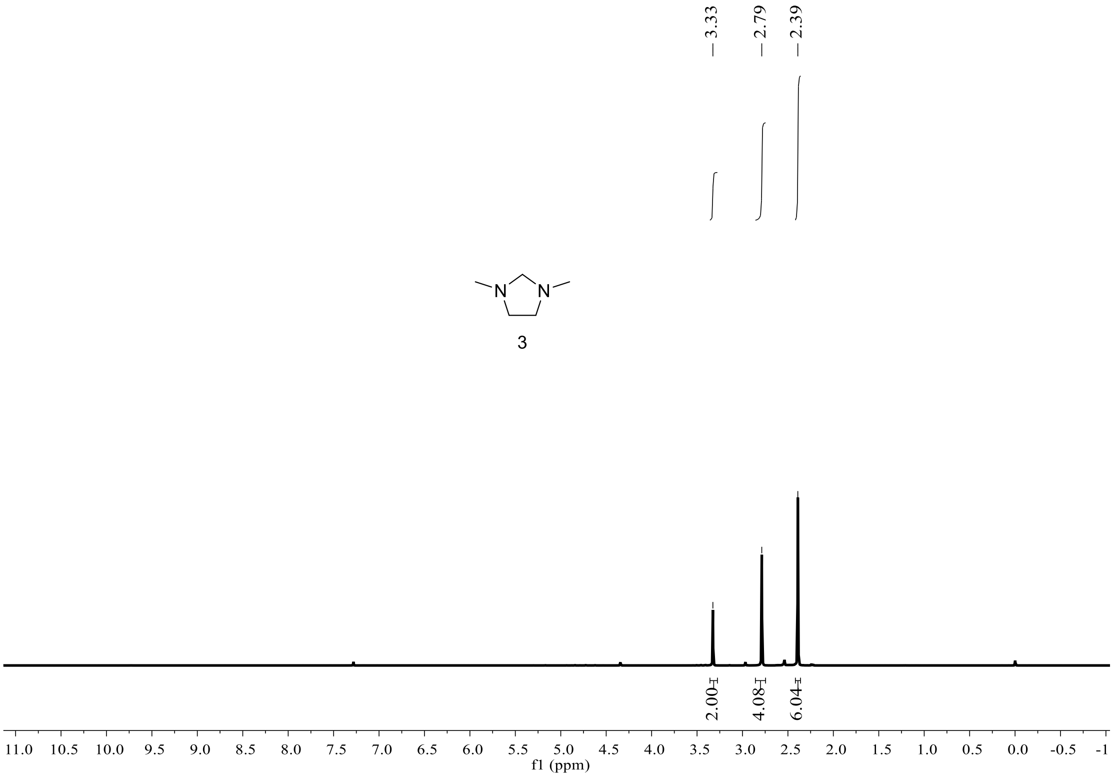

Supplementary Figure 47. ^{13}**C NMR (100 MHz, CDCl$_3$) spectrum of 3**

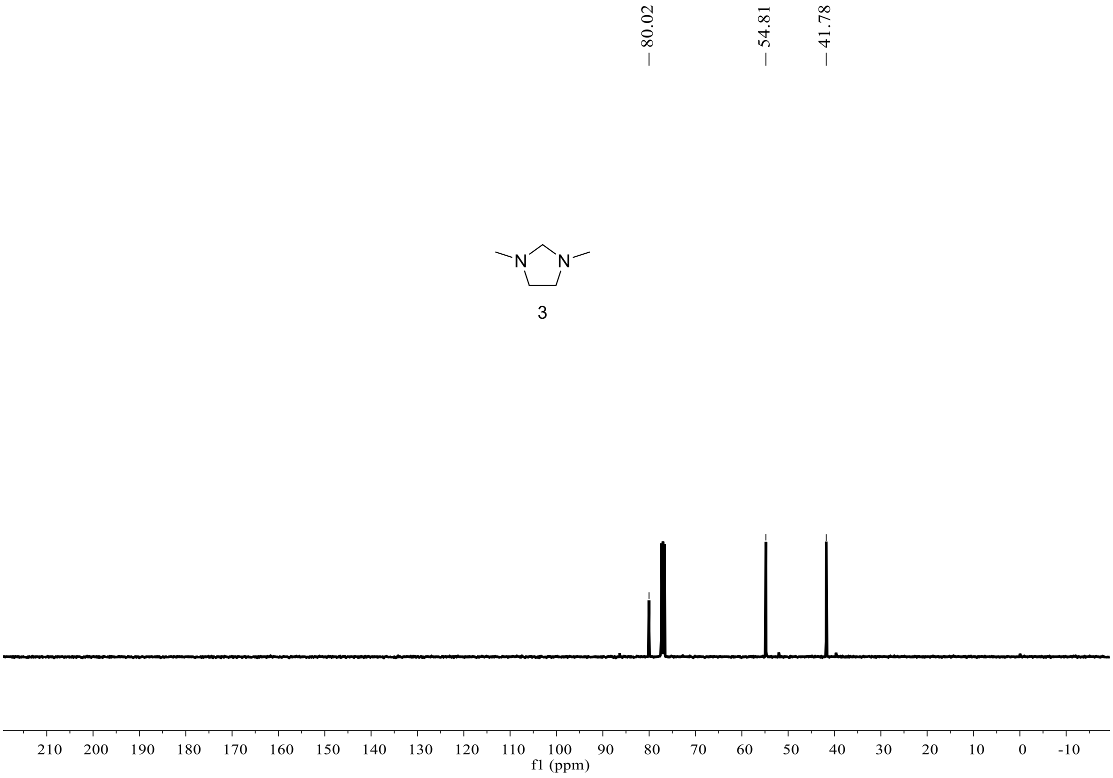

Supplementary tables

Supplementary Table 1. Optimization of manganese pincer precatalysts

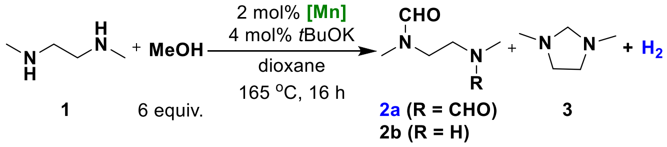

Entry	[Mn]	Conv. [%]	2a [%]	2b [%]	3 [%]	H_2 [%]
1	**I**	0	0	0	0	trace
2	**II**	0	0	0	0	trace
3	**III**	0	0	0	0	trace
4	**IV**	0	0	0	0	trace
5	**V**	>99	31	24	45	48(>99.9%)
6	**VI**	**>99**	**86**	**9**	**5**	**92(95.5%)**

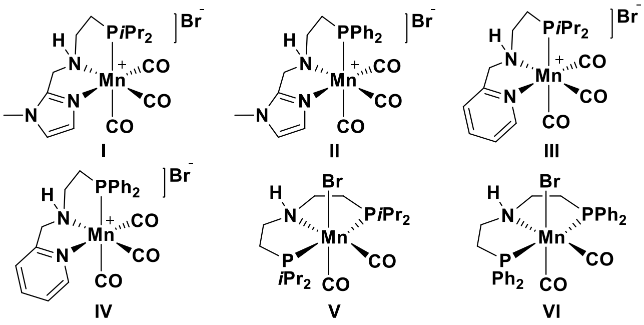

Reaction conditions: **1** (0.25 mmol), MeOH (6equvi.), **Mn** (0.005 mmol, 2 mol%), *t*BuOK (0.01 mmol, 20 mol%) and dioxane (0.4 mL) were reacted at 165 ℃ for 16 h. The conversion and yield were determined by NMR and GC with 1,1,2,2-tetrachloroethane as the internal standard

Supplementary Table 2. Optimization of base and solvent

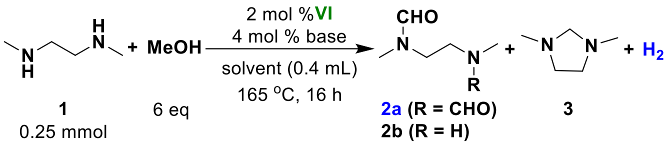

Entry	Base	Solvent	Conv. [%]	2a [%]	2b [%]	3 [%]	H_2 [%]
1	*t*BuOK	dioxane	>99	86	9	5	92(95.5%)
2	*t*BuONa	dioxane	>99	30	22	46	48(>99.9%)
3	KOMe	dioxane	>99	85	10	5	90(98.5%)
4	KOH	dioxane	>99	71	14	15	80(99.8%)
5	EtONa	dioxane	>99	26	23	56	43(>99.9%)
6	*t*BuOK	P-xylene	>99	87	8	5	91(96.5%)
7	*t*BuOK	toluene	>99	87	9	4	91(97.1%)
8	*t*BuOK	mesitylene	>99	83	9	6	89(97.2%)

Reaction conditions: **1** (0.25 mmol), MeOH (6equvi.), **Mn-VI** (0.005 mmol), base (0.01 mmol) and solvent (0.4 ml) were reacted at 165 ℃ for 16 h. The conversion and yield were determined by NMR and GC with 1,1,2,2-tetrachloroethane as the internal standard

Supplementary Table 3. Optimization of other parameters

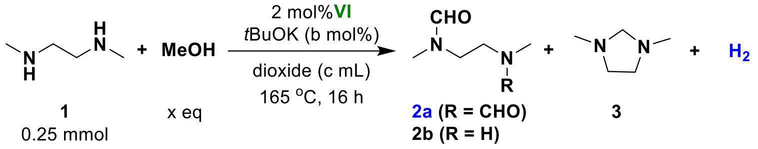

Entry	x [eq]	b [mol]	c [mL]	Conv. [%]	2a [%]	2b [%]	3 [%]	H_2 [%]
1	6	4	0.4	>99	86	9	5	92(95.5%)
2	8	4	0.4	>99	86	5	8	89(94.8%)
3	4	4	0.4	>99	80	13	7	85(96.7%)
4	6	8	0.4	>99	83	9	8	88(88.7%)
5	6	6	0.4	>99	86	9	5	90(92.3%)
6	6	2	0.4	>99	82	8	10	86(98.3%)
7	6	4	0.2	>99	77	8	15	82(96.4%)
8	6	4	0.6	>99	86	11	3	90(93.5%)
9	6	4	1	>99	77	17	5	88(90.2%)
10[a]	6	4	0.4	>99	76	14	7	84(97.8%)
11[b]	6	4	0.4	>99	97	<1	2	**98(98.7%)**
12[c]	6	4	0.4	>99	93	5	2	**90(>99.9%)**

Reaction conditions: **1** (0.25 mmol), **VI** (2 mol%), MeOH, *t*BuOK and dioxane were reacted at 165 °C for 16 h. The conversion and yield were determined by NMR and GC with 1,1,2,2-tetrachloroethane as the internal standard

[a]The reaction temperature was 150 °C

[b]After reacting for 2 h, the reaction was cooled down to r.t. and the evolved gas was released from the system. And then the reaction was heated to 165 °C again and reacted for another 6 h

[c]VI (1 mol%) and *t*BuOK (4 mol%) were used. After reacting for 2 h, the reaction was cooled down to r.t. and the evolved gas was released from the system. And then **VI** (1 mol%) was added before the reaction was heated to 165 °C again and reacted for another 6 h

Supplementary Table 4. Volume optimization of dehydrogenation solvents

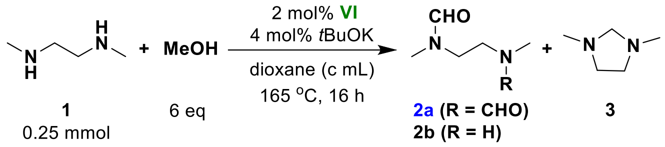

Entry	c [mL]	Conv. [%]	2a [%]	2b [%]	3 [%]
1	0.4	>99	97	<1	2
2	0.3	>99	90	7	2
3	0.2	>99	84	10	6
4	0.1	90	55	21	10
5	0	61	16	26	14

Reaction conditions:**1** (0.25 mmol), MeOH (6 equvi.), **Mn-VI** (0.005 mmol, 2 mol%), *t*BuOK (0.01 mmol, 4 mol%) and dioxane were reacted at 165 ℃, After 2 h, the reaction mixture was cooled to room temperature and the evolved gas was released from the system. The temperature was then increased to 165 ℃ and the reaction was performed for a further 6 h. The conversion and yield were determined by NMR and GC with 1,1,2,2-tetrachloroethane as the internal standard

Supplementary Table 5. Optimization of manganese pincer precatalysts

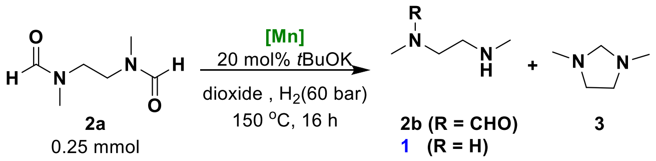

Entry	[Mn]	Conv. [%]	2b [%]	1 [%]	3 [%]
1	**I**	>99	37	49	12
2	**II**	>99	56	44	0
3	**III**	>99	20	79	0
4	**IV**	>99	0	99	0
5	**V**	>99	53	38	0
6	**VI**	>99	46	47	7

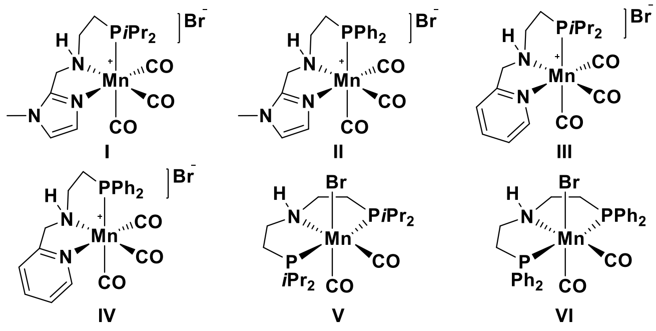

Reaction conditions: **2a** (0.25 mmol), **Mn** (0.005 mmol), *t*BuOK (0.05 mmol) and dioxane (1 mL) were reacted at 150 ℃ under 60 bar H_2 for 16 h. The conversion and yield were determined by GC and NMR with 1,1,2,2-tetrachloroethane as the internal standard

Supplementary Table 6. Optimization of base and solvent

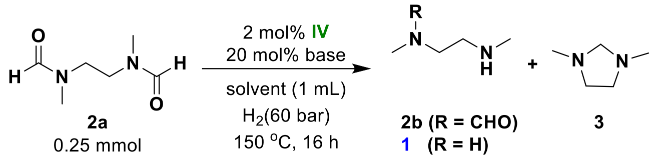

Entry	Base	Solvent	Conv. [%]	2b [%]	1 [%]	3 [%]
1	*t*BuOK	1,4-dioxane	>99	0	99	0
2	*t*BuONa	1,4-dioxane	>99	20	65	5
3	KOMe	1,4-dioxane	94	48	35	10
4	KOH	1,4-dioxane	97	41	48	6
5	EtONa	1,4-dioxane	>99	19	73	7
6	*t*BuOK	P-xylene	>99	12	84	0
7	*t*BuOK	toluene	>99	19	81	0
8	*t*BuOK	mesitylene	>99	13	77	6

Reaction conditions: **2a** (0.25 mmol), **Mn-IV** (0.005 mmol), base (0.05 mmol) and solvent (1 mL) were reacted at 150 ℃ under 60 bar H_2 for 16 h. The conversion and yield were determined by GC and NMR with 1,1,2,2-tetrachloroethane as the internal standard

Supplementary Table 7. Optimization of other parameters

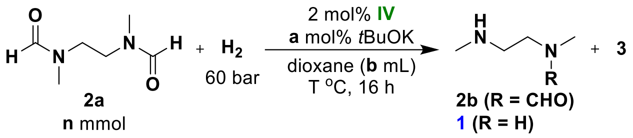

Entry	n [mmol]	a [mol%]	b [mL]	T [°C]	2b [%]	1 [%]	3 [%]
1	0.25	20	1	150	0	99	0
2[a]	0.25	20	1	150	37	61	0
3	0.25	20	1	130	0	99	0
4	0.25	20	1	110	0	99	0
5	0.25	20	1	90	50	38	8
6	0.25	10	1	110	15	76	0
7	0.5	10	2	110	0	99	0
8	**0.5**	**2.5**	**2**	**110**	**0**	**99**	**0**
9	**0.5**	**2.5**	**1**	**110**	**0**	**99**	**0**
10	**0.5**	**2.5**	**0.4**	**110**	**0**	**99**	**0**

Reaction conditions: **2a**, **Mn-IV**, *t*BuOK and dioxane were reacted at given temperature under 60 bar H_2 for 16 h. The conversion and yield were determined by GC and NMR with 1,1,2,2-tetrachloroethane as the internal standard .[a]40 bar

Supplementary Table 8. Optimization of using **[Mn]-VI** as catalyst

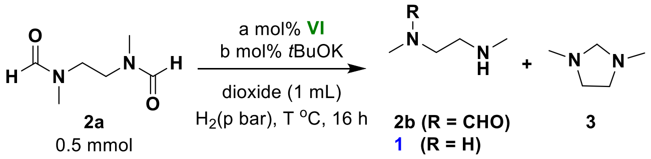

Entry	a [mol]	b [mol]	p [bar]	T [°C]	Conv. [%]	2b [%]	1 [%]	3 [%]
1	2	5	60	160	>99	46	46	0
2	2	10	60	160	>99	54	34	0
3	2	5	80	160	>99	43	55	0
4	2	5	80	180	>99	9	91	0
5	2	2.5	80	180	>99	16	84	0
6[a]	2	5	80	180	>99	16	82	0
7[b]	2	5	80	180	>99	23	77	0
8	3	5	80	180	>99	5	94	0
9[c]	3	5	80	180	7	7	0	0

Reaction conditions: **2a**, **Mn-VI**, *t*BuOK and dioxane were reacted at given temperature under given pressure for 16 h. The conversion and yield were determined by GC and NMR with 1,1,2,2-tetrachloroethane as the internal standard

[a]dioxane (0.5 mL)

[b]dioxane (2 mL)

[c]**[Mn]-VII** (3 mol%)

Supplementary Table 9. Volume optimization of hydrogenation solvents catalyzed by **[Mn]-IV**

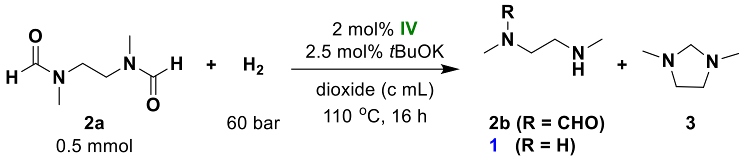

Entry	c [mL]	Conv. [%]	2b [%]	1 [%]	3 [%]
1	2	>99	0	>99	0
2	1	>99	0	>99	0
3	0.8	>99	0	>99	0
4	0.6	>99	0	>99	0
5	0.4	>99	0	>99	0
6	0.3	>99	0	92	5
7	0.2	>99	<1	72	25

Reaction conditions: **2a** (0.5 mmol), **Mn-IV** (0.01 mmol, 2 mol%), *t*BuOK (0.0125 mmol) and dioxane were reacted at 110 ℃ under 60 bar H_2 for 16 h. The conversion and yield were determined by GC and NMR with 1,1,2,2-tetrachloroethane as the internal standard

Supplementary Table 10. Volume optimization of hydrogenation solvents catalyzed by **[Mn]- VI**

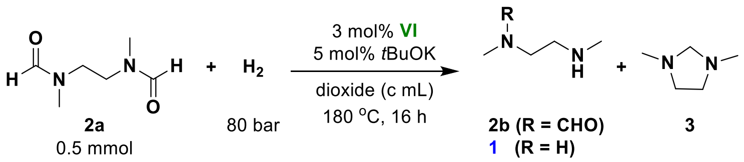

Entry	c [mL]	Conv. [%]	2b [%]	1 [%]	3 [%]
1	1	>99	5	94	0
2	0.8	>99	6	93	0
3	0.6	>99	5	94	0
4	0.4	>99	5	94	0
5	0.3	>99	2	82	14

Reaction conditions: **2a** (0.5 mmol), **Mn-VI** (0.015 mmol, 3 mol%), *t*BuOK (0.025 mmol) and dioxane were reacted at 180 ℃ under 80 bar H_2 for 16 h. The conversion and yield were determined by GC and NMR with 1,1,2,2-tetrachloroethane as the internal standard

Supplementary Table 11. Homogenity test for the dehydrogenation reaction catalyzed by **[Mn]-VI**

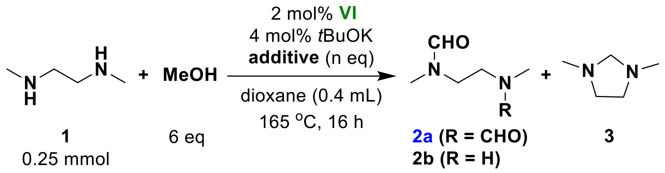

Entry	additive	n [eq]	Conv. [%]	2a [%]	2b [%]	3 [%]
1	**--**	--	>99	86	9	5
2	**Hg**	A drop	>99	84	7	8
3	**PMe_3**	0.1	>99	86	9	5
4	**PMe_3**	0.5	>99	86	9	5
5	**PMe_3**	1	>99	85	10	4
6	**PPh_3**	0.1	>99	85	10	5
7	**PPh_3**	0.5	>99	85	9	5
8	**PPh_3**	1	>99	84	9	6

Reaction conditions: **1** (0.25 mmol), MeOH (6equvi.), **Mn-VI** (0.005 mmol, 2 mol%), *t*BuOK (0.01 mmol, 4 mol%), dioxane (0.4 mL) and phosphines or mercury (equiv. respect to **Mn-VI**) were reacted at 165 ℃ for 16 h. The conversion and yield were determined by NMR and GC with 1,1,2,2-tetrachloroethane as the internal standard

Supplementary Table 12. Homogenity test for the hydrogenation reaction catalyzed by **[Mn]-IV**

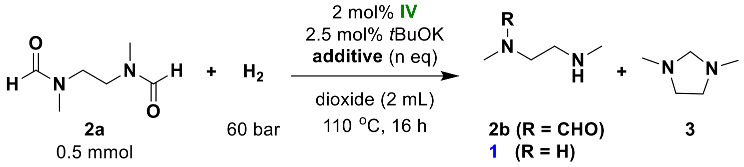

Entry	additive	n [eq]	Conv. [%]	2b [%]	1 [%]	3 [%]
1	--	--	>99	0	>99	0
2	**Hg**	A drop	>99	0	>99	0
3	**PMe_3**	0.1	>99	0	>99	0
4	**PMe_3**	0.5	>99	0	>99	0
5	**PMe_3**	1	>99	0	>99	0
6	**PPh_3**	0.1	>99	0	>99	0
7	**PPh_3**	0.5	>99	0	>99	0
8	**PPh_3**	1	>99	0	>99	0

Reaction conditions: **2a** (0.5 mmol), **Mn-IV** (0.01 mmol, 2 mol%), *t*BuOK (0.0125 mmol, 2.5 mol%), dioxane (2 mL) and phosphines or mercury (equiv. respect to **Mn-IV**) were reacted at given temperature under given pressure for 16 h. The conversion and yield were determined by GC and NMR with 1,1,2,2-tetrachloroethane as the internal standard

Supplementary Table 13. Homogenity test for the hydrogenation reaction catalyzed by **[Mn]-VI**

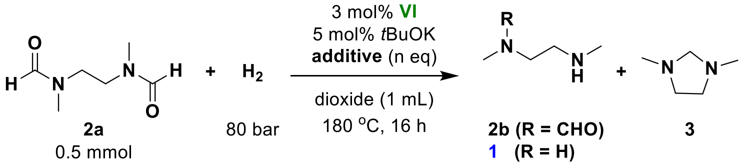

Entry	additive	n [eq]	Conv. [%]	2b [%]	1 [%]	3 [%]
1	**--**	--	>99	5	94	0
2	**Hg**	A drop	>99	7	92	0
3	**PMe_3**	0.1	>99	6	93	0
4	**PMe_3**	0.5	>99	5	94	0
5	**PMe_3**	1	>99	5	94	0
6	**PPh_3**	0.1	>99	6	94	0
7	**PPh_3**	0.5	>99	4	95	0
8	**PPh_3**	1	>99	4	95	0

Reaction conditions: **2a** (0.5 mmol), **Mn-VI** (0.015 mmol, 3 mol%), *t*BuOK (0.025 mmol, 5 mol%),, dioxane (1 mL) and phosphines or mercury (equiv. respect to **Mn-VI**) were reacted at given temperature under given pressure for 16 h. The conversion and yield were determined by GC and NMR with 1,1,2,2-tetrachloroethane as the internal standard

Supplementary Table 14. Dehydrogenation reaction of (Table 2, entry 11).

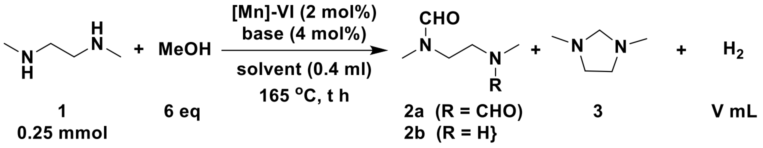

Entry	t (h)	V (mL)	Purity (%)	Conv. [%]	2a [%]	2b [%]	3 [%]
1	The first 2 h	16.4	98.1	83	57	24	2
2	The next 6 h	7.6	99.9	> 99	97	< 1	2

Supplementary Table 15. Dehydrogenation reaction of (Table 2, entry 12)

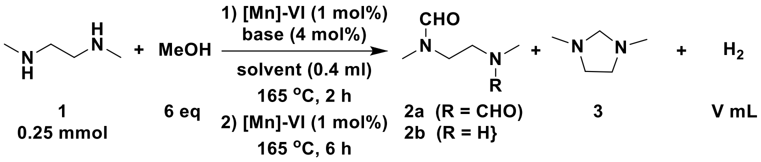

Entry	t (h)	V (mL)	Purity (%)	Conv. [%]	2a [%]	2b [%]	3 [%]
1	The first 2 h	10.6	>99.9	57	32	22	2
2	The next 6 h	11.0	>99.9	> 99	93	5	2

Supplementary Table 16. Dehydrogenation reaction of (Fig 2)

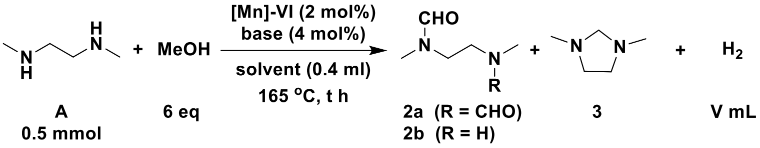

Entry	t (h)	V (mL)	Purity (%)	Conv. [%]	2a [%]	2b [%]	3 [%]
1	The first 4 h	30.8	97.9	83	46	35	2
2	The next 6 h	14.4	98.6	> 99	94	4	2

Supplementary Table 17. Crystallographic data of **[Mn]-VII**

CCDC	Formula	Crystal system	Space group	*a*[Å]	b[Å]	c[Å]
1949549	$C_{31}H_{31}BrMnNO_2P_2$	orthorhombic	-P 2ac 2ab	17.2957(2)	17.2051 (2)	19.2508(2)
α[º]	β[º]	γ[º]	Cell volume[Å3]	Z	Crystal size[mm^3]	Density[Mg/m^3]
90.00	90.00	90.00	5728.54(11)	8	0.25 x 0.15 x 0.05	1.499
T[K]	Theta range[º]	Reflections collected				
173.00(10)	4.291 to 66.599	5057				

Supplementary methods

General Information: The synthesis of complexes **I**, **III**, **IV** and **V** were prepared according to previous reports[1,2]. Air and moisture sensitive reactions were carried out in glovebox or in over-dried glassware sealed with rubber septa using standard schlenk techniques. Solvents used in moisture and oxygen sensitive reactions were firstly degassed by three freeze-pump-thaw cycles and then distilled under Ar protection after dehydration (dioxane were dried over sodium metal). Deuterated solvents were ordered from Cambridge Isotope Laboratories. All chemicals were purchased from commercial sources with purity over 95% and used without further purification. NMR spectra were received using a Bruker 400 MHz spectrometer. Chemical shifts are reported in ppm relative to the deuterated solvent. GC analysis were carried out on SHIMADAZU GC 2010 PLUS system. (column: SH-Rtx-200, 30 m x 0.25 mm x 0.25 μm). The gas mixtures were analyzed using a SHIMADAZU GC 2010 PLUS system. (column: SHINCARBON ST, 2.0 m x 1.0 mmI.D.) equipped with a BID detector. High resolution exact mass measurements (HRMS) were performed on Thermo SCIENTIFIC Q EXACTIVE.

Synthesis of 2-(diphenylphosphaneyl)ethan-1-amine

In a 100 mL Schlenk flask, a solution of $HPPh_2$ (1.86 g, 10 mmol) in 20 mL of anhydrous THF was cooled to 0 ℃, and treated dropwise with *n*BuLi solution in hexane (2.5 M, 4.4 mL, 11 mmol). The reaction mixture was turned into deep red color immediately and further stirred at room temperature for 2 h. And then the solution was again cooled to 0 ℃ and *N*, *N*-bis(trimethylsilyl)-2-chloroethanamine (2. 45 g, 11 mmol) was added dropwise. The mixture was warmed to room temperature and then refluxed at 75 ℃ for 12 hours. Afterwards the reaction mixture was cooled to 0 ℃. Water (5 mL) was added, followed by 2.0 M H_2SO_4 solution (6 mL). After stirring for 1 hour at 0 ℃, a solution of 4.0 M NaOH solution (7 mL) was then added, and the mixture was stirred for another 0.5 h. The organic layer was separated and the aqueous phase was extracted with Et_2O (3 × 20 mL). Combined organic layer was dried (Na_2SO_4), filtered and evaporated in vacuo to give 2-(diphenylphosphaneyl)ethan-1-amine as a pale yellow oil crude product without further purification (2.06 g, 90 %).

Synthesis of 2-(diphenylphosphaneyl)-*N*-((1-methyl-1*H*-imidazol-2-yl)methyl)ethan-1-amine L-II

2-(diphenylphosphaneyl)ethan-1-amine (0.29 g, 1.0 mmol) in 6 mL of THF was added to 1-methyl-1*H*-imidazole-2-carbaldehyde (0.11 g, 1.0 mmol) in 6 mL of THF. The mixture was stirred for 1 h at room temperature, then evaporated. The above oil residue was dissolved in 12 mL of toluene and was slowly added 1.5 M solution of DIBAL (1.5 M, 1.2 mL, 1.8 mmol) in toluene. The product solution was stirred for 2 h at 0 ℃, and then quenched with 10 mL of water and extracted with Et_2O (3 × 15 mL). The combined organic layer was dried over Na_2SO_4, filtered and concentrated under vacuum. And the residue was purified by chromatography on silica gel to give pure desirable product as a pale yellow oil (0.21 g, 65 %).

^{1}H NMR (400 MHz, $CDCl_3$) δ 7.41 (m, 4 H), 7.35 – 7.29 (m, 6 H), 6.91 (d, *J* = 1.3 Hz, 1 H), 6.80 (d, *J* = 1.3 Hz, 1 H), 3.82 (s, 2 H), 3.64 (s, 3 H), 2.86 – 2.74 (m, 2 H), 2.31 – 2.23 (m, 2 H), 1.77 (b, 1 H). ^{13}C NMR (100 MHz, $CDCl_3$) δ 146.32, 138.32 (d, *J* = 12.4 Hz), 132.66 (d, *J* = 18.7 Hz), 128.58,

128.42 (d, J = 6.7 Hz), 127.12, 121.16, 46.26 (d, J = 20.3 Hz), 45.56, 32.72, 28.90 (d, J = 12.4 Hz). ^{31}P NMR (162 MHz, $CDCl_3$) δ -20.76 (s).

HRMS (ESI) calcd. for $C_{19}H_{22}N_3P$ $[M+H]^+$: 324.1624; found: 324.1610.

Synthesis of bis(2-(diphenylphosphaneyl)ethyl)amine L-VI

In a 100 mL Schlenk flask, a solution of $HPPh_2$ (1.86 g, 10 mmol) in 20 mL of anhydrous THF was cooled to 0 ℃, and treated dropwise with *n*BuLi solution in hexane (2.5 M, 4.4 mL, 11 mmol). The reaction mixture was turned into deep red color immediately and further stirred at room temperature for 2 h. And then the solution was again cooled to 0 ℃ and *N*, *N*-bis(2-chloroethyl)-1,1,1-trimethylsilanamine (0. 96 g, 4.5 mmol) was added dropwise. The mixture was warmed to room temperature and then refluxed at 75 °C for 12 hours. Afterwards the reaction mixture was cooled to 0 ℃. Water (8 mL) was added, followed by 2.0 M H_2SO_4 solution (2.5 mL). After stirring for 1 hour at 0 ℃, a solution of 4.0 M NaOH solution (2.8 mL) was then added, and the mixture was stirred for another 0.5 h. The organic layer was separated and the aqueous phase was extracted with Et_2O (3 × 20 mL). Combined organic layer was dried (Na_2SO_4), filtered and evaporated in vacuo, the residue was purified by chromatography on silica gel to give bis(2-(diphenylphosphaneyl)ethyl)amine product as a white solid (1.10 g, 56 %).

^{1}H NMR (400 MHz, $CDCl_3$) δ 7.39 (td, J = 7.2, 3.1 Hz, 8 H), 7.34 – 7.28 (m, 12 H), 2.84 (dt, J = 10.7, 6.4 Hz, 4 H), 2.49 – 2.32 (m, 4 H). ^{13}C NMR (100 MHz, $CDCl_3$) δ 136.06 (d, J = 12.2 Hz), 132.53 (d, J = 19.1 Hz), 129.03, 128.66 (d, J = 6.7 Hz), 44.17 (d, J = 26.4 Hz), 23.78 (d, J = 16.1 Hz). ^{31}P NMR (162 MHz, $CDCl_3$) δ -20.87 (s).

HRMS (ESI) calcd. for $C_{28}H_{29}NP_2$ $[M+H]^+$: 442.1848; found: 442.1842.

Synthesis of 2-(diphenylphosphaneyl)-N-(2-(diphenylphosphaneyl)ethyl)-N-methylethan-1-amine L-VII

A 50 mL Schlenk flask was charged with diphenylphosphane (1.50 g, 8 mmol) and degassed dry THF (10 mL) and cooled to –78 ℃. To this cooled solution was added nBuLi (4 mL, 2.5 M in hexane, 10 mmol) dropwise and then the resulting solution was reacted for 1 h. After that the solution was cooled to –78 ℃ again. Another 50 mL Schlenk flask was charged with $MeN(CH_2CH_2Cl)_2 \cdot HCl$ (0.62 g, 3.2 mmol) and THF (10 mL). It is then cooled to –78 ℃. To the suspension was added nBuLi (1.5 mL, 2.5 M in hexane, 3.75 mmol) dropwise and stirred for 0.5 h while warming to room temperature. The resulting mixture was cooled to –78 ℃ and added dropwise to the flask containing the lithium phosphide solution at -78 ºC. The mixture was then stirred at reflux overnight. The solution was subsequently cooled to room temperature before removing the solvent under vacuum. The reaction mixture was quenched with 5 mL of water and extracted with Et_2O (3 ×5mL). The combined organic layer was dried with Na_2SO_4, filtered and concentrated under vacuum. After chromatography on silica-gel column (CH2Cl2/MeOH=100/1 to 50/1), the corresponding ligand was obtained as yellowish liqiud (0.71 g, 49%).

^{1}H NMR (400 MHz, $CDCl_3$) δ 7.39 (td, J = 7.2, 3.1 Hz, 8 H), 7.33 – 7.29 (m, 12 H), 2.48 (m, 4 H), 2.24 (s, 3 H), 2.36 – 2.12 (m, 4 H). ^{13}C NMR (100 MHz, $CDCl_3$) δ 138.44 (d, J = 12.2 Hz), 132.70 (d, J = 19.1 Hz), 128.58(s), 128.45 (s),128.38 (s), 53.38 (d, J = 27.3Hz), 41.75 (s) , 25.75 (d, J = 12.1Hz). ^{31}P NMR (162 MHz, $CDCl_3$) δ -19.79 (s).

HRMS (ESI) calcd. for $C_{29}H_{31}NP_2$ $[M+H]^+$: 456.2004; found: 456.1988.

Synthesis of {Mn(CO)$_3$(2-(diphenylphosphaneyl)-*N*-[(1-methyl-1*H*-imidazol-2-yl)methyl]ethan-1-amine}Br [Mn]-II

In a 50 mL Schlenk flask, a solution of [MnBr(CO)$_5$] (274 mg, 1 mmol) in 10 mL of toluene was added to a solution of 2-(diphenylphosphaneyl)-*N*-[(1-methyl-1*H*-imidazol-2-yl)methyl]ethan-1-amine **L-II** (340 mg, 1.05 mmol) in 5 mL of toluene at room temperature. The resulting solution was then heated to 110 ^{o}C for 12 hours. After that, yellow precipitate was formed, collected by filtration, and washed with toluene (1 mL) for three times. Then the filter cake was dried in vacuum to give faint yellow powder product (433 mg, 80%).

^{1}H NMR (400 MHz, DMSO-d_6) δ 7.76 (t, *J* = 7.6 Hz, 2 H), 7.55 (m, 3 H), 7.45 – 7.34 (m, 5 H), 7.07 (s, 1 H), 6.42 (s, 1 H), 4.32 (d, *J* = 16.8 Hz, 1 H), 4.08 (d, *J* = 16.8 Hz, 1 H), 3.56 (s, 3 H), 3.14 (m, 1 H), 2.87 – 2.69 (m, 1 H), 2.27 (m, 2 H). ^{13}C NMR (100 MHz, DMSO-d_6) δ 149.77, 132.67, 132.26, 131.95, 131.85, 131.74, 131.40, 131.04, 130.92, 129.74 (d, *J* = 9.5 Hz), 129.41 (d, *J* = 9.3 Hz), 128.08, 125.24, 54.90 (d, *J* = 11.4 Hz), 49.69, 34.71, 22.48 (d, *J* = 22.6 Hz). ^{31}P NMR (162 MHz, DMSO-d_6) δ 63.97 (s).

HRMS (ESI) calcd. for $C_{22}H_{22}BrMnN_3O_3P$ $[M-Br]^+$: 462.0774; found: 462.0775.

Synthesis of Bromodicarbonyl(bis(2-(diphenylphosphaneyl)ethyl)amine)manganese [Mn]-VI

In a 50 mL Schlenk flask, a solution of [MnBr(CO)$_5$] (274 mg, 1 mmol) in 10 mL of toluene was added to a solution of bis(2-(diphenylphosphaneyl)ethyl)amine **L-VI** (463 mg, 1.05 mmol) in 5 mL of toluene at room temperature. The resulting solution was then heated to 110 ^{o}C for 20 hours. After that, it was cooled to room temperature and concentrated in vacuo. Hexane (20 mL) was added to the reaction Schlenk flask and then the suspension was filtered. The crude precipitate was extracted with dichloromethane and the resulted filtrate solution was concentrated under reduced pressure and dried to afford the complex **[Mn]-VI** as a yellow powder (391 mg, 62%).

^{1}H NMR (400 MHz, DCM-d_2) δ 7.90 (m, *J* = 5.9 Hz, 4 H), 7.59 (m, 4 H), 7.52 – 7.22 (m, 12 H), 3.67 (m, 2 H), 3.54 (br, 1 H), 3.27 (m, 2 H), 2.77 (m, 2 H), 2.41 (m, 2 H). ^{13}C NMR (100 MHz, DCM-d_2) δ 138.03 (t, *J* = 18.9 Hz), 135.46 (t, *J* = 18.8 Hz), 133.88 (t, *J* = 4.8 Hz), 130.68 (t, *J* = 5.0 Hz), 130.32, 129.66, 129.06 (t, *J* = 4.2 Hz), 128.75 (t, *J* = 4.5 Hz), 53.05 (t, *J* = 4.8 Hz), 28.50 (t, *J* = 8.8 Hz). ^{31}P NMR (162 MHz, DCM-d_6) δ 69.65 (s).

Synthesis of {MnBr(CO)$_2$[Me-N(CH$_2$CH$_2$P(Ph)$_2$)$_2$]} [Mn]-VII

2-(diphenylphosphaneyl)-*N*-(2-(diphenylphosphaneyl)ethyl)-*N*-methylethan-1-amine (160 mg, 0.35 mmol, 1.06 equivalents) was added to pentacarbonylbromomanganese (I) (92 mg, 0.33 mmol, 1.0 equivalents) in a 50 mL Schlenk flask at ambient temperature. Degassed toluene (10 mL) was added and the mixture was heated to reflux and kept at that temperature for 24 h. After that, it was cooled to room temperature and concentrated in vacuo. The crude material was dissolved in methylene chloride, filtered to remove insoluble material and the product precipitated by addition of n-pentane, collected by filtration and washed with n-hexane and to give the desired product as a yellow powder (175 mg, 0.27 mmol, 82% yield).

^1H NMR (400 MHz, DCM-d_2) δ 7.89 – 7.76 (m, 8 H), 7.49 – 7.35 (m, 12 H), 3.79 – 3.72 (m, 2 H), 3.24 – 3.14 (m, 2 H), 3.01 – 2.93 (m, 2 H), 2.81 – 2.71 (m, 2 H), 2.67 (s, 3 H). ^{13}C NMR (101 MHz, DCM-d_2) δ 132.34 (t, J = 5.0 Hz), 131.50 (t, J = 5.1 Hz), 129.50 (s), 129.03 (s), 128.53 (t, J = 4.4 Hz), 127.78 (t, J = 3.9 Hz), 59.08 (t, J = 4.4 Hz), 49.94 (s) , 27.77 (t, J = 8.8 Hz). ^{31}P NMR (162 MHz, DCM-d_6) δ 69.06 (s).
HRMS (ESI) calcd. for $C_{31}H_{31}BrMnNO_2P_2$ $[M-Br]^+$: 566.1205; found: 566.1205.
Crystal structure was shown in **Supplementary Figure 8**.

General procedure for the optimization of manganese pincer precatalysts

All dehydrogenation experiments were carried out in a 25 mL pressure seal tube. In the argon atmosphere glovebox, manganese pincer precatalysts (0.005 mmol, 2 mol%), *t*BuOK (0.01 mmol, 4 mol%) dioxane (0.4 mL), *N*,*N*'-dimethylethylenediamine **1** (0.25 mmol) and MeOH (6 equiv., 1.5 mmol) were added sequentially to the seal tube equipped with a magnetic stir bar, The reaction mixture was stirred at 165 ℃ for 16 hours and cooled to room temperature. After the gas was released, the conversion of **1** was determined by NMR and the yield of products **2a**, **2b** and **3** was determined by GC wiith 1,1,2,2-tetrachloroethane as the internal standard.

General procedure for the optimization of base and solvent

All dehydrogenation experiments were carried out in a 25 mL pressure seal tube. In the argon atmosphere glovebox, **[Mn]-VI** (0.005 mmol, 2 mol%), base (0.01 mmol, 4 mol%) solvent (0.4 mL), *N,N'*-dimethylethylenediamine **1** (0.25 mmol) and MeOH (6 equiv., 1.5 mmol) were added sequentially to the seal tube equipped with a magnetic stir bar, The reaction mixture was stirred at 165 ℃ for 16 hours and cooled to room temperature. After the gas was released, the conversion of **1** was determined by NMR and yield of products **2a**, **2b** and **3** was determined by GC wiith 1,1,2,2-tetrachloroethane as the internal standard.

General procedure for the optimization of other parameters

All dehydrogenation experiments were carried out in a 25 mL pressure seal tube. In the argon atmosphere glovebox, **[Mn]-VI** (2 mol%), *t*BuOK, dioxane, *N*,*N*'-dimethylethylenediamine **1** (0.25 mmol) and MeOH were added sequentially to the seal tube equipped with a magnetic stir bar, The reaction mixture was stirred at given temperature for 16 hours and cooled to room temperature. After the gas was released, the conversion of **1** was determined by NMR and the yield of products **2a**, **2b** and **3** was determined by GC wiith 1,1,2,2-tetrachloroethane as the internal standard.

General procedure for volume optimization of dehydrogenation solvents

All dehydrogenation experiments were carried out in a 25 mL pressure seal tube. In the argon atmosphere glovebox, **[Mn]-VI** (2 mol%), *t*BuOK (0.01 mmol, 4 mol%), dioxane, *N*,*N*'-dimethylethylenediamine **1** (0.25 mmol) and MeOH (6 equvi.) were added sequentially to the seal tube equipped with a magnetic stir bar, The reaction mixture was stirred at 165 ℃, After 2 h, the

reaction mixture was cooled to room temperature and the evolved gas was released from the system. The temperature was then increased to 165 ℃ and the reaction was performed for a further 6 h and cooled to room temperature. After the gas was released, the conversion of **1** was determined by NMR and the yield of products **2a**, **2b** and **3** was determined by GC wiith 1,1,2,2-tetrachloroethane as the internal standard.

General procedure for the optimization of manganese pincer precatalysts

All hydrogenation experiments were carried out in a Parr Instruments 4560 series autoclave (300 mL) containing an alloy plate with wells for seven 4 mL glass vials. In the argon atmosphere glovebox, *N*,*N*'-(Ethane-1,2-diyl)bis(*N*-methylformamide) **2a** (0.25 mmol), manganese precatalysts (0.005 mmol, 2 mol%), *t*BuOK (0.05 mmol, 20 mol%) and dioxane (1 mL) were added sequentially to the vial equipped with a magnetic stir bar, which was capped with a septum threaded with a syringe. The vial was placed in the alloy plate, which was then placed to the predried autoclave. Once sealed, the autoclave was purged 3 times with hydrogen, then pressurized to 60 bar H_2 and heated at 150 ℃ for 16 h. After reaction, the autoclave was cooled to 0 ℃, depressurized. The conversion and yield were determined by GC and NMR with 1,1,2,2-tetrachloroethane as the internal standard.

General procedure for the optimization of base and solvent

All hydrogenation experiments were carried out in a Parr Instruments 4560 series autoclave (300 mL) containing an alloy plate with wells for seven 4 mL glass vials. In the argon atmosphere glovebox, *N*,*N*'-(Ethane-1,2-diyl)bis(*N*-methylformamide) **2a** (0.25 mmol), **[Mn]-IV** (0.005 mmol, 2 mol%), base (0.05 mmol, 20 mol%) and solvent (1 mL) were added sequentially to the vial equipped with a magnetic stir bar, which was capped with a septum threaded with a syringe. The vial was placed in the alloy plate, which was then placed to the predried autoclave. Once sealed, the autoclave was purged 3 times with hydrogen, then pressurized to 60 bar H_2 and heated at 150 ℃ for 16 h. After reaction, the autoclave was cooled to 0 ℃, depressurized. The conversion and yield were determined by GC and NMR with 1,1,2,2-tetrachloroethane as the internal standard.

General procedure for the optimization of other parameters

All hydrogenation experiments were carried out in a Parr Instruments 4560 series autoclave (300 mL) containing an alloy plate with wells for seven 4 mL glass vials. In the argon atmosphere glovebox, *N*,*N*'-(Ethane-1,2-diyl)bis(*N*-methylformamide) **2a**, **[Mn]-IV** (0.005 mmol, 2 mol%), *t*BuOK and dioxane were added sequentially to the vial equipped with a magnetic stir bar, which was capped with a septum threaded with a syringe. The vial was placed in the alloy plate, which was then placed to the predried autoclave. Once sealed, the autoclave was purged 3 times with hydrogen, then pressurized to 60 bar H_2 and heated at given temperature for 16 h. After reaction, the autoclave was cooled to 0 ℃, depressurized. The conversion and yield were determined by GC and NMR with 1,1,2,2-tetrachloroethane as the internal standard.

General procedure for the optimization of using [Mn]-VI as catalyst

All hydrogenation experiments were carried out in a Parr Instruments 4560 series autoclave (300 mL)

containing an alloy plate with wells for seven 4 mL glass vials. In the argon atmosphere glovebox, *N,N'*-(Ethane-1,2-diyl)bis(*N*-methylformamide) **2a** (0.5 mmol), **[Mn]-VI**, *t*BuOK and dioxane were added sequentially to the vial equipped with a magnetic stir bar, which was capped with a septum threaded with a syringe. The vial was placed in the alloy plate, which was then placed to the predried autoclave. Once sealed, the autoclave was purged 3 times with hydrogen, then pressurized to given pressure and heated at given temperature for 16 h. After reaction, the autoclave was cooled to 0 ℃, depressurized. The conversion and yield were determined by GC and NMR with 1,1,2,2-tetrachloroethane as the internal standard.

General procedure for volume optimization of hydrogenation solvents catalyzed by [Mn]-IV and [Mn]-VI

All hydrogenation experiments were carried out in a Parr Instruments 4560 series autoclave (300 mL) containing an alloy plate with wells for seven 4 mL glass vials. In the argon atmosphere glovebox, *N,N'*-(Ethane-1,2-diyl)bis(*N*-methylformamide) **2a** (0.5 mmol), **[Mn]-IV** (0.01 mmol, 2 mol%) or **[Mn]-VI** (0.015 mmol, 3 mol%), *t*BuOK and dioxane were added sequentially to the vial equipped with a magnetic stir bar, which was capped with a septum threaded with a syringe. The vial was placed in the alloy plate, which was then placed to the predried autoclave. Once sealed, the autoclave was purged 3 times with hydrogen, then pressurized to given pressure and heated at given temperature for 16 h. After reaction, the autoclave was cooled to 0 ℃, depressurized. The conversion and yield were determined by GC and NMR with 1,1,2,2-tetrachloroethane as the internal standard.

Homogenity test with ligand poision experiments

All dehydrogenation experiments were carried out in a 25 mL pressure seal tube. In the argon atmosphere glovebox, **[Mn]-VI** (0.005 mmol, 2 mol%), base (0.01 mmol, 4 mol%) dioxane (0.4 mL), *N,N'*-dimethylethylenediamine **1** (0.25 mmol), MeOH (6 equiv., 1.5 mmol) and phosphines or mercury (equiv. respect to complex **VI**) were added sequentially to the seal tube equipped with a magnetic stir bar, The reaction mixture was stirred at 165 ℃ for 16 hours and cooled to room temperature. After the gas was released, the conversion of **1** was determined by NMR and yield of products **2a**, **2b** and **3** was determined by GC wiith 1,1,2,2-tetrachloroethane as the internal standard.

All hydrogenation experiments were carried out in a Parr Instruments 4560 series autoclave (300 mL) containing an alloy plate with wells for seven 4 mL glass vials. In the argon atmosphere glovebox, *N,N'*-(Ethane-1,2-diyl)bis(*N*-methylformamide) **2a** (0.5 mmol), **[Mn]-** (2 mol%) or **[Mn]-VI** (3 mol%), *t*BuOK, dioxane and phosphines or mercury (equiv. respect to complex **IV** or **VI**) were added sequentially to the vial equipped with a magnetic stir bar, which was capped with a septum threaded with a syringe. The vial was placed in the alloy plate, which was then placed to the predried autoclave. Once sealed, the autoclave was purged 3 times with hydrogen, then pressurized to given pressure and heated at given temperature for 16 h. After reaction, the autoclave was cooled to 0 ℃, depressurized. The conversion and yield were determined by GC and NMR with 1,1,2,2-tetrachloroethane as the internal standard.

The collectioin of evolved gas and analysis of gas phase components (Table 2, entry 11)

The dehydrogenation experiments were carried out in a 25 mL pressure seal tube. In the argon atmosphere glovebox, **[Mn]-VI** (0.005 mmol, 2 mol%), *t*BuOK (0.01 mmol, 4 mol%), dioxane (0.4 mL) *N*,*N*'-dimethylethylenediamine **1** (0.25 mmol) and MeOH (6 equiv., 1.5 mmol) were added sequentially to the seal tube equipped with a magnetic stir bar, The reaction mixture was stirred at 165 ℃ for 2 hours and cooled to 20 ℃, the evolved gas was released from the system, which was collected and measured by the following apparatus **(Supplementary Figure 12)**. After releasing the gas, the reaction was heated to 165 ºC again and reacted for another 6 h. After cooling down, the evolved gas during the reaction process was collected and measured again at 20 ℃. The result of GC analysis was shown below. (The yield of H_2 was caculated on the basis of maxium H_2 evolution with respect to 100% conversion of **1** to **2a**. The molar volume of hydrogen at 20 ℃ and 1 atm pressure is taken as 24.1 L.)

GC analysis of the gas phase: GC conditions: Packed Column. Inlets: 100 ℃; Detector: BID 200 ℃; Carrier Gas: He; Flow: 51.4 mL/min; Oven: 35 ℃, hold 2 min; 5 ℃ /min to 80 ℃, hold 5 min.

The collectioin of evolved gas and analysis of gas phase components (Table 2, entry 12)

The dehydrogenation experiments were carried out in a 25 mL pressure seal tube. In the argon atmosphere glovebox, **[Mn]-VI** (0.0025 mmol, 1 mol%), *t*BuOK (0.01 mmol, 4 mol%), dioxane (0.4 mL) *N*,*N*'-dimethylethylenediamine **1** (0.25 mmol) and MeOH (6 equiv., 1.5 mmol) were added sequentially to the seal tube equipped with a magnetic stir bar, The reaction mixture was stirred at 165 ℃ for 2 hours and cooled to 20 ℃, the evolved gas was released from the system, which was collected and measured by the above apparatus **(Supplementary Figure 12)**. After releasing the gas, **[Mn]-VI** (0.0025 mmol, 1 mol%) was added, the reaction was heated to 165 ºC again and reacted for another 6 h. After cooling down, the evolved gas during the reaction process was collected and measured again at 20 ℃. The result of GC analysis was shown below. (The yield of H_2 was caculated on the basis of maxium H_2 evolution with respect to 100% conversion of **1** to **2a**. The molar volume of hydrogen at 20 ℃ and 1 atm pressure is taken as 24.1 L.)

Procedure for Reversible interconversion between 1/methanol and 2a by Mn-catalyzed hydrogenation and dehydrogenation（Fig 2）

The dehydrogenation experiments were carried out in a 25 mL pressure seal tube. In the argon atmosphere glovebox, **[Mn]-VI** (0.01 mmol, 2 mol%), *t*BuOK (0.02 mmol, 4 mol%), dioxane (0.4 mL) *N*,*N*'-dimethylethylenediamine **1** (0.5 mmol) and MeOH (6 equiv., 3 mmol) were added sequentially to the seal tube equipped with a magnetic stir bar, The reaction mixture was stirred at 165 ºC for 4 hours and cooled to 20 ℃, the evolved gas was released from the system, which was collected and measured by the above apparatus **(Supplementary Figure 12)**. After releasing the gas, the reaction was heated to 165 ºC again and reacted for another 6 h. After cooling down, the evolved gas during the reaction process was collected and measured again at 20 ℃. The result of GC analysis was shown below. (The yield of H_2 was caculated on the basis of maxium H_2 evolution with respect to 100% conversion of **1** to **2a**. The molar volume of hydrogen at 20 ℃ and 1 atm pressure is taken as 24.1 L.)

Condition A: the dehydrogenation reaction mixture was transfered to a Parr Instruments 4560 series

autoclave (300 mL) containing an alloy plate with a 4 mL glass vials in glovebox. Then, **[Mn]-IV** (0.01 mmol, 2 mol%), *t*BuOK (0.0125 mmol, 2.5 mol%) were also added sequentially to the vial, which was capped with a septum equipped with a syringe. The vial was placed in the alloy plate, which was then placed to the predried autoclave. Once sealed, the autoclave was purged 3 times with hydrogen, then pressurized to 60 bar H_2 and heated at 110 °C for 16 h. After reaction, the autoclave was cooled to 0 ℃, depressurized. The conversion and yield were determined by GC and NMR with 1,1,2,2-tetrachloroethane as the internal standard.

Condition B: the dehydrogenation reaction system was transfer to a Parr Instruments 4560 series autoclave (300 mL) containing an alloy plate with a 4 mL glass vials in glovebox. Meanwhile, **[Mn]-VI** (0.015 mmol, 3 mol%), *t*BuOK (0.025 mmol, 5 mol%) and dioxane (0.6 mL) were also added sequentially to the vial, which was capped with a septum equipped with a syringe. The vial was placed in the alloy plate, which was then placed to the predried autoclave. Once sealed, the autoclave was purged 3 times with hydrogen, then pressurized to 80 bar H_2 and heated at 180 °C for 16 h. After reaction, the autoclave was cooled to 0 ℃, depressurized. The conversion and yield were determined by GC and NMR with 1,1,2,2-tetrachloroethane as the internal standard.

Procedure for Mn-catalyzed dehydrogenation of MeOH (Fig 4a)

The reaction was carried out in a 25 mL pressure seal tube. In the argon atmosphere glovebox, **[Mn]-VI** (0.005 mmol), *t*BuOK (0.01 mmol) dioxane (0.4 mL) and MeOH (1.5 mmol) were added sequentially to the seal tube equipped with a magnetic stir bar, The reaction mixture was stirred at 165 °C for 16 hours and cooled to 20 ℃, the evolved gas was released from the system, which was collected and measured by the above apparatus **(Supplementary Figure 12)**. The result of GC analysis and NMR spetra was shown below. (The molar volume of hydrogen at 20 ℃ and 1 atm pressure is taken as 24.1 L.)

Synthesis of *N*,*N*'-(Ethane-1,2-diyl)bis(*N*-methylformamide) (2a)[3]

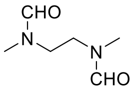

Ethyl formate (4.0 mL, 50.0 mmol) and *N*,*N*'-dimethylethane-1,2-diamine (538.1 μL , 5.0 mmol) were mixed to form a solution and the mixture was heated at 90 ℃ for 24 h. After cooling down to room temperature, the reaction mixture was washed with 1 mL of 2M HCl and extracted with DCM (3×5 mL). The organic layers were combined and dried over anhydrous Na_2SO_4. Evaporation of the solvent and the product was obtained by recrystallization of ether and ethyl acetate as white solid (446.4 mg, 62%). ^{1}H NMR (400 MHz, $CDCl_3$) δ8.18 – 7.87 (m, 2H), 3.66 – 3.31 (m, 4H), 3.10 – 2.78 (m, 6H). ^{13}C NMR (100 MHz, $CDCl_3$) δ 162.89, 162.68, 162.28, 162.28, 47.27, 46.17, 42.25, 40.29, 35.08, 34.33, 29.76, 29.60.

Synthesis of *N*-Methyl-*N*-[2-(methylamino)ethyl]formamide (2b)

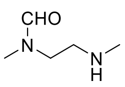

Ethyl formate (4 mL, 50.0 mmol) and *N*,*N*'-dimethylethane-1,2-diamine (5.4 mL , 0.5 mol) were combined to form a solution and the mixture was heated at 70 ℃ for 16 h. After cooled to room temperature, the product was purified by vacuum distillation and isolated as a yellow oil (2.6 g, 45 % yield). ^{1}H NMR (400 MHz, $CDCl_3$) δ8.05 – 8.00 (m, 1H), 3.44 –

3.28 (m, 2H), 2.96 – 2.83 (m, 3H). 2.78 – 2.62 (m, 2H), 2.42 – 2.39 (m, 3H). ^{13}C NMR (100 MHz, $CDCl_3$) δ 162.91, 162.84, 49.30, 48.97, 48.69, 43.89, 36.27, 36.21, 35.00, 29.59. HRMS (ESI) calcd. for $C_5H_{13}N_2O$ ($[M+H]^+$) 117.10224, Found 117.10277.

Synthesis of 1,3-Dimethylimidazolidine (3)[4]

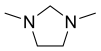

In a 100 mL Schlenk flask, paraformaldehyde (5.00 g, 16.60 mmol) and diethyl ether (20 mL) were added to the *N*,*N*'-dimethylethane-1,2-diamine (16.90 mmol). The mixture was stirred for 16 h under 40 ℃, the product was purified by atmospheric distillation and isolated as a colorless oil (85 % yield). ^{1}H NMR (400 MHz, $CDCl_3$) δ3.33 (s, 2H), 2.79 (s, 4H), 2.39 (s, 6H). ^{13}C NMR (100 MHz, $CDCl_3$) δ 80.02, 54.81, 41.78.

Supplementary References

1. Fu, S., Shao, Z., Wang, Y. & Liu, Q. Manganese-Catalyzed Upgrading of Ethanol into 1-Butanol. *J. Am. Chem. Soc.* **139**, 11941-11948 (2017).
2. Zubar, V., Lebedev, Y., Azofra, L.M., Cavallo, L., El-Sepelgy, O. & Rueping, M. Hydrogenation of CO2-Derived Carbonates and Polycarbonates to Methanol and Diols by Metal–Ligand Cooperative Manganese Catalysis. *Angew. Chem. Int. Ed.* **57**, 13439-13443 (2018).
3. Luan, Y.-X., Zhang, T., Yao, W.-W., Lu, K., Kong, L.-Y., Lin, Y.-T. & Ye, M. Amide-Ligand-Controlled Highly para-Selective Arylation of Monosubstituted Simple Arenes with Arylboronic Acids. *J. Am. Chem. Soc.* **139**, 1786-1789 (2017).
4. Denk, M.K., Gupta, S., Brownie, J., Tajammul, S. & Lough, A.J. C–H Activation with Elemental Sulfur: Synthesis of Cyclic Thioureas from Formaldehyde Aminals and S8. *Chem. Eur. J.* **7**, 4477-4486 (2001).
